# USP20 is a predictor of poor prognosis in colorectal cancer and associated with lymph node metastasis, immune infiltration and chemotherapy resistance

**DOI:** 10.3389/fonc.2023.1023292

**Published:** 2023-02-16

**Authors:** RuiRi Jin, ZhiPeng Luo, Jun-Li, Qing Tao, Peng Wang, XueSheng Cai, LongZhou Jiang, ChunYan Zeng, YouXiang Chen

**Affiliations:** ^1^ Department of Gastroenterology, Digestive Disease Hospital, The First Affiliated Hospital of Nanchang University, Nanchang, China; ^2^ Department of Abdominal Tumor Surgery, Jiangxi Cancer Hospital, Nanchang, China; ^3^ Jiangxi Provincial Key Laboratory of Interdisciplinary Science, Nanchang University, Nanchang, China

**Keywords:** colorectal cancer (CRC), ubiquitin specific peptidase 20 (USP20), lymph node metastasis, infiltrating immune, bioinformatics analysis, predictive models

## Abstract

**Background:**

Colorectal cancer (CRC) is a highly prevalent malignancy with a poor prognosis. USP20 can support progression of variety of tumors. USP20 was shown to promote breast tumor metastasis, and proliferation of oral squamous carcinoma cells. However, the role of USP20 in CRC remains unclear.

**Methods:**

We used bioinformatics to analyze the expression and prognosis of USP20 in pan-cancer and explore the relationship between USP20 expression and immune infiltration, immune checkpoints, and chemotherapy resistance in CRC. The differential expression and prognostic role of USP20 in CRC was validated by qRT-PCR and immunohistochemistry. Cox univariate and multivariate analyses were performed to assess risk factors for poor prognosis of CRC, and new prognostic prediction models were constructed and evaluated by decision curve analysis (ROC) and receiver operating characteristic (DCA). USP20 was overexpressed in CRC cell lines to explore the effect of USP20 on the functionalities of CRC cells. Enrichment analyses were used to explore the possible mechanism of USP20 in CRC.

**Results:**

The expression of USP20 was lower in CRC tissues than adjacent normal tissues. Compared with low USP20 expression patients, CRC patients with high USP20 expression level had shorter OS. Correlation analysis showed that USP20 expression was associated with lymph node metastasis. Cox regression analysis revealed USP20 as an independent risk factor for poor prognosis in CRC patients. ROC and DCA analyses showed that the performance of the newly constructed prediction model was better than the traditional TNM model. Immune infiltration analysis shown that USP20 expression is closely associated with T cell infiltration in CRC. A co-expression analysis showed that USP20 expression was positively correlated with several immune checkpoint genes including ADORA2A, CD160, CD27 and TNFRSF25 genes and positively associated with multiple multi-drug resistance genes such as MRP1, MRP3, and MRP5 genes. USP20 expression positively correlated with the sensitivity of cells to multiple anticancer drugs. Overexpression of USP20 enhanced the migration and invasive ability of CRC cells. Enrichment pathway analyses showed the USP20 may play a role *via* the Notch pathway, Hedgehog pathway and beta-catenin pathway.

**Conclusion:**

USP20 is downregulated in CRC and associated with prognosis in CRC. USP20 enhances CRC cells metastasis and is associated with immune infiltration, immune checkpoints, and chemotherapy resistance.

## Introduction

Colorectal cancer (CRC) is the third most common cancer and the third most common cause of cancer-related death in the United States. The American Cancer Society estimates that there will be over 151,030 new cases of CRC in the United States in 2022, with an estimated 54,250 deaths (1). Currently, surgery is the most effective treatment for CRC, however it is a curative treatment for only early-stage CRC patients. In recent years, neoadjuvant chemotherapy has substantially improved CRC outcomes by reducing tumor burden, increasing the rates of rectal preservation, improving the 5-year survival rate, and providing an opportunity for advanced-stage cancer patients to undergo surgery to improve prognosis (2–4). However, postoperative tumor recurrence is still an issue for CRC patients, leading to cancer progression and even death. Therefore, further clarifying the pathophysiological mechanism of CRC, exploring new therapeutic targets, and reducing the postoperative recurrence of CRC are of great significance to improve patient prognosis.

Ubiquitination is one of the main pathways that regulates the stability of intracellular proteins and involves the modification of target proteins by ubiquitin. Ubiquitination is a dynamic and reversible process that plays a role in numerous biological processes including the cell cycle, proliferation, apoptosis, differentiation, metastasis and other biological processes. Multiple studies have demonstrated an important function of ubiquitination in cancer. Previous reports showed that ROR-γt ubiquitination inhibits IL-17 mediated colon inflammation and tumorigenesis (5). In non-small cell lung cancer, circIGF2BP3 inhibits CD8+ T-cell responses to facilitate tumor immune evasion by promoting the deubiquitination of PD-L1 (6). SPOP-mediated ubiquitination and degradation of PDK1 suppresses AKT kinase activity and oncogenic functions (7). Deubiquitinating enzymes are vital to maintain the ubiquitination balance. Compared with the research on ubiquitinase enzymes, the research on deubiquitinases is scarce.

Ubiquitin specific peptidase 20 (USP20) is a member of the peptidase C19 family and the encoding gene is located on chromosome 9. USP20 was first identified as a deubiquitinating enzyme in 2002 (8). Studies have shown that USP20 is involved in the regulation of autophagy, inflammatory response, viral immune response, and cholesterol biosynthesis (9–12). Several reports have examined the role of USP20 in cancer, and the results have been controversial. Some researchers found that USP20 promotes the metastasis of breast cancer (13). However, another study showed that USP20 suppresses the malignant characteristics of gastric cancer cells (14). These results suggest an important role of USP20 in cancer. However, its potential function in CRC has not been investigated.

In this study, we explored the expression and possible mechanism of USP20 in CRC using bioinformatics analysis and we preliminarily verified our results through cytological experiments. We analyzed clinical samples to explore the prognostic value and clinical relevance of USP20 in CRC. Our results may help provide new insights into the mechanisms of CRC mechanisms and the development of new therapeutic approaches for CRC.

## Materials and methods

### Patients and samples

A total of 92 formalin-fixed paraffin-embedded pairs of tumor and adjacent normal tissue samples were collected from CRC patients undergoing surgery at Jiangxi Cancer Hospital (Nanchang, China) from 2017 to 2019. The specimens were stained by hematoxylin and eosin (HE) and observed by multiple pathologists to confirm the diagnosis of CRC. Regular telephone interviews were conducted after surgery. The clinicopathological characteristics of patients are shown in **Table 1**. An additional independent set of samples was obtained from 23 CRC patients who underwent surgery at Jiangxi Cancer Hospital between 2021 and 2022. These samples were stored in liquid nitrogen for quantitative real-time PCR( qRT-PCR ) analysis. The clinicopathological characteristics of this patient cohort are shown in **Table 2**. All patients provided written informed consent. This study conformed to the Declaration of Helsinki and was approved by the Institutional Ethics Committee of Jiangxi Cancer Hospital (Nanchang, Jiangxi, China, ethics approval no. 2022ky061).

**Table 1:** Association between USP20 protein expression and clinicopathologic characteristics of patients with CRC in the first study cohort (n=92).

			USP20 protein level		
Characterstic	Level	Overall	Low	High	P-value
n		92	41	51	
Status (%)	Alive	72 (78.3)	39 (95.1)	33 (64.7)	0.001
	Dead	20 (21.7)	2 (4.9)	18 (35.3)	
Sex (%)	M ale	66 (71.7)	27 (65.9)	39 (76.5)	0.373
	Fem ale	26 (28.3)	14 (34.1)	12 (23.5)	
Age (year) (%)	<60	46 (50.0)	16 (39.0)	30 (58.8)	0.093
	60	46 (50.0)	25 (61.0)	21 (41.2)	
Primary tumor location (%)	Rightcobn	40 (43.5)	19 (46.3)	21 (41.2)	0.119
	Rectum	5 (5.4)	0 (0.0)	5 9.8)	
	Leftcoln	47 (51.1)	22 (53.7)	25 (49.0)	
T (%)	T1/T2	9 (9.8)	6 (14.6)	3 (5.9)	0.293
	T3/T4	83 (90.2)	35 (85.4)	48 (94.1)	
N %)	NO	46 (50.0)	31 (75.6)	15 (29.4)	<0.001
	N 1/N2	46 (50.0)	10 (24.4)	36 (70.6)	
M (%)	MO	88 (95.7)	40 (97.6)	48 (94.1)	0.771
	M 1	4 (4.3)	1 (2.4)	3 (5.9)	
Degree of differentiation (%)	Poor	25 (27.2)	7 (17.1)	18 (35.3)	0,09
	Well	1 (1.1)	1 (2.4)	0 (0.0)	
	Moderate	66 (71.7)	33 (80.5)	33 (64.7)	
Pathological type (%)	High-grade intraepithelial neoplasia	1 (1.1)	1 (2.4)	0 (0.0)	0.037
	adenocarcinoma	79 (85.9)	39 (95.1)	40 (78.4)	
	Signet ring cell carcinomas	3 (3.3)	0 (0.0)	3 (5.9)	
	Mucinous Adenocarcinoma.	9 (9.8)	1 (2.4)	8 (15.7)	
Tumor size (%)	≥5cm	69 (75.0)	30 (73.2)	39 (76.5)	0.904
	<5cm	23 (25.0)	11 (26.8)	12 (23.5)	
Perineural invasion (%)	Postive	25 (27.2)	10 (24.4)	15 (29.4)	0.762
	Negative	67 (72.8)	31 (75.6)	36 (70.6)	
Lymphovascular invasion (%)	Postive	38 (41.3)	14 (34.1)	24 (47.1)	0.3
	Negative	(58.7)	27 (65.9)	27 (52.9)	
AJCC stage (%)	I + II	(50.0)	30 (73.2)	16 (31.4)	<0.001
	III + IV	(50.0)	11 (26.8)	35 (68.6)	
Adjuvant chemotherapy (%)	No	(23.9)	8 (19.5)	14 (27.5)	0.521
	Yes	70 (76.1)	33 (80.5)	37 (72.5)	

**Table 2:** Clinicopathological characteristics of the second study cohort (n=23) for assessing USP20 mRNA level.

Characteristic	N
Sex	
Female	10
Male	13
Age (years)	
<60	11
≥60	12
Tumor location	
Rectum	9
Colon	14
Degree of differentiation	
Well + moderate	13
Poor	10
Tumor size (cm)	
<5	14
≥5	9
Local invasion	
pT1-T2	3
pT3-T4	20
Lymph node metastasis	
N0	14
N1+N2	9
TNM stage	
I + II	14
III+IV	9
Perineural invasion	
Postive	8
Negative	15
Lymphovascular invasion	
Postive	12
Negative	11

### Data and software availability

All bioinformatics data were downloaded from The Cancer Genome Atlas (TCGA) (https://ca-ncergenome.nih.gov/), GTEx database (https://gtexportal.org/), Gene Expression Omnibus (GEO) (https://www.ncbi.nlm.nih.gov/geo), and Therapeutically Applicable Research To Generate Effective Treatments (TARGET) (https://ocg.cancer.gov/programs/target) databases. The differential expression of USP20 levels between tumor and normal tissues across TCGA database was analyzed by the TIMER online system (https://cistrome.shinyapps.io/timer/) (15). R 4.1.0 was used to integrate the original data and verify the results analyzed by the website database. Data from TCGA, GTEx, and TARGET were downloaded using the online tool xiantao-love (https://www.xiantao.love/).

### Analysis of differentially expressed genes (DEGs)

CRC samples from TCGA were divided into high USP20 expression and low USP20 expression groups using the median expression value of USP20. Differential gene expression between different groups of samples were analyzed by the DESeq2 package (16). We used adjusted P-values to avoid false-positive results. The screening criteria for DEGs genes in this study were set as |log2(FC)| >1, P.adj< 0.05. The results of the differential gene expression analysis were presented by volcano plots using the ggplot2 package.

### Enrichment analysis

To explore the target and possible mechanism of USP20 in CRC, ClusterProfiler package was used for DEG enrichment analysis, including GO Enrichment and KEGG Enrichment (17). The results of USP20 single gene differential analysis showed that the USP20 was mainly accompanied by the low expression of DEGs. We selected the top 100 significantly downregulated DEGs for GO and KEGG analysis. To further observe the effect of USP20 on CRC, the enrichment of Hallmark pathways related to USP20 expression were analyzed by GSEA (18).

### Survival analysis

Univariate and multivariable Cox analyses were used to analyze the relationship between USP20 expression and CRC patient overall survival (OS). Kaplan–Meier (KM) curves were used to demonstrate the difference in OS between patients with different USP20 expression levels. Clinical prediction models were constructed on the basis of Cox regression results. Clinical usefulness as well as net benefit of model was estimated by decision curve analysis (DCA). The prognostic performance of the different models was assessed by receiver operating characteristic (ROC) curve analysis.

### Immunological correlation analysis

Using the immune cell scores of CRC in the TIMER database, we analyzed the correlation between gene expression and immune cell scores. Furthermore, the correlation between USP20 and CD4+ T cell subsets was calculated using ssGSEA method implemented by R package GSVA (19). We collected more than 40 common immune checkpoint genes and performed molecular correlation analysis with USP20 in TCGA.

### Immunohistochemistry

CRC tissues and adjacent normal tissues were warmed at 70°C for 1.5 h, dewaxed sequentially with xylene and anhydrous ethanol, heated at high temperature in a microwave oven for 15 min, and incubated in citrate buffer for antigen retrieval. After natural cooling, the tissues were incubated with primary antibody overnight at 4°C. The next day, after washing with PBS, the tissues were incubated with secondary antibody for 30 min at room temperature. The tissues were stained with DAB reagent (TransGen Biotech, Beijing, China) and the nuclei were stained with hematoxylin. Staining was scored following the methods described in a previous article (20). Two histopathologists were blindly assigned to review the slides and score the staining. The staining was considered as positive when the score was ≥6.

### Immunofluorescence

Tissue was dewaxed and antigen-repaired following the same steps described above. After antigen repair, the tissue was permeabilized with 0.2% Triton X-100, following by blocking in 5% BSA and incubation overnight with the primary antibody. The next day, the tissues were washed with PBS and incubated with secondary antibody under light-proof conditions. After sealing with nail polish, the tissue was observed under a confocal microscope.

### Cell culture and transfection

Human CRC cell lines (HCT116, DLD-1 and SW480) and a normal colon mucosal epithelial cell line (NCM460) were purchased from the Cell Bank of the Chinese Academy of Sciences. Cells were cultured in RPMI-1640 medium (Gibco, Grand Island, NY, USA) supplemented with 10% FBS (Gibco, USA), 100 U/ml penicillin, and 50 mg/ml streptomycin at 37 °C in a humidified atmosphere containing 5% CO_2_. SW480 cells were transfected with the USP20 overexpression plasmid or control plasmid using Lipo3000 Transfection Reagent (Invitrogen, USA). The USP20 plasmid and control plasmid were constructed by Public Protein/Plasmid Library (Nanjing, China). For knockdown of USP20, three siRNAs targeting USP20 were constructed by Public Protein/Plasmid Library (Nanjing, China). The above 3 siRNAs are named siRNA-1-208, siRNA-2-1310 and siRNA-3-1816 respectively. Sequences of the siRNAs were as follows: siRNA-1-208(CCAUAGGAGAGGUGACCAATT), siRNA-2-1310(GGACAAUGAUGCUCACCUATT), siRNA-3-1816(CUGAUGAGUUAAAGGGUGATT), negative control siRNA (UUCUCCGAACGUGUCACGUTT). SW480 cells were transfected with control siRNA or with siRNAs against USP20, by using Lipo3000 Transfection Reagent (Invitrogen, USA).

### Quantitative real-time PCR

Total RNA was extracted with TRIzol reagent (ET111-01,TransGen Biotech, Beijing), phase-separated with chloroform, and precipitated using isopropanol. RNA concentration was measured with a NanoDrop 2000 spectrophotometer (Thermo Scientific, Wilmington, DE, USA). Reverse transcription of total RNA was performed using PrimeScript RT Master Mix (RR036A, Takara, Kusatsu, Japan) following the manufacturer’s instructions. qRT-PCR was performed with kit reagents (RR420A, Takara, Kusatsu, Japan) to detect USP20 mRNA levels. The sequences of primers for qRT-PCR were as follows: USP20 forward: 5′- CCTCACCTTGACTCCATAGGA -3′ and reverse: 5′-CCCATAGGTTTGGTCCGGT -3′; CD4 forward: 5′-TGCCTCAGTATGCTGGCTCT-3′ and reverse: 5′-GAGACCTTTGCCTCCTTGTTC-3′;ADORA2A forward: 5′-CGCTCCGGTACAATGGCTT -3′ and reverse: 5′-TTGTTCCAACCTAGCATGGGA -3′;CD160 forward: 5′-GCTGAGGGGTTTGTAGTGTTT-3′ and reverse: 5′-GTGTGACTTGGCTTATGGTGA-3′;CD27 forward: 5′-CAGAGAGGCACTACTGGGCT-3′ and reverse: 5′-CGGTATGCAAGGATCACACTG-3′;CD200 forward: 5′-AAGTGGTGACCCAGGATGAAA and reverse: 5′-AGGTGATGGTTGAGTTTTGGAG; GAPDH forward: 5′- CCATGTTCGTCATGGTGTG -3′ and reverse: 5′- GGTGCTAAGCAGTTGTGGTG -3′.

### Western blot

Total protein was extracted from CRC cells using radioimmunoprecipitation assay (RIPA) buffer (Solarbio Life Science, Beijing, China), which was mixed with protease inhibitor on precooled plates. The details of the experimental conditions are described in the Methods section and in our previous paper (21). Throughout this study, primary antibodies targeting the proteins are listed as follows: GAPDH (1:1000, TransGen Biotech, Beijing,China), β-actin (1:1000, TransGen Biotech, Beijing,China), and USP20 (17491-1-AP, 1:1000, Proteintech, China).

### Cell proliferation assay

The Cell Counting Kit-8 (40203E; Yeasen, Shanghai, China) was used for cell proliferation assays. Cells were seeded in 96-well plates at a density of 1×10^3^ cells per well, incubated at 37°C for 24 h, and transfected with the USP20 overexpression plasmid or control plasmid. After cultivation for 24 h, 48 h, or 72 h, 10 µl CCK-8 reagent was added to each well and cells were incubated for 2 h. The optical density (OD) values were read at 450 nm.

### Cell migration and invasion assays

Transwell assays were used to determine the invasion and migration of CRC cells. SW480 cells were transfected with the USP20 overexpression plasmid or control plasmid. After transfection, 10×10^5^ SW480 cells were seeded into the upper chamber of a Transwell system (8 µm pore size, Corning, USA) with or without Matrigel (BD Biosciences, USA). Then, 800 µl medium with 20% FBS was added to the lower chamber. The Transwell chambers were incubated for 48 h. Transwell chambers were then placed in 4% paraformaldehyde and stained with 0.5% crystal violet for 30 min. Stained cells were quantified using a microscope at 200× magnification. We randomly selected five visual fields, recorded the number of cells in each field, and calculated the mean value.

### Anti-tumor drug sensitivity analysis

We accessed the NCI-60 database at the CellMiner website and downloaded gene expression data for 60 different cancer lines of cells and data for 263 antitumor drugs for Pearson correlation analysis (https://discover.nci.nih.gov/cellminer) (22). The gene expression and anti-tumor drug data are shown in **Table S1**-**S2**.

### Statistical analysis

R (4.10) software was used for statistical analysis. Data are presented as means ± SD of the mean. The data for two group comparisons were first subjected to normality tests. If the data sets fit a normal distribution, unpaired, two-tailed t-test was used; if not, nonparametric Mann–Whitney and Wilcoxon signed-rank tests were used. Differences among more than two groups were evaluated by one-way ANOVA. All statistical tests were 2-sided; P < 0.05 indicated statistical significance.

## Results

### Bioinformatics analysis for pan-cancer analysis of USP20

**Figure 1:**
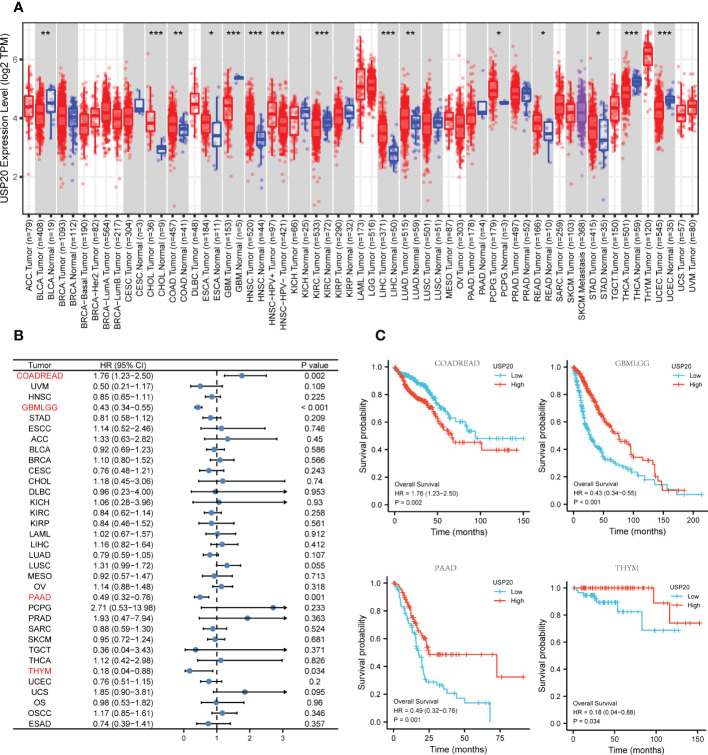
Pan-cancer analysis of USP20; **(A)** USP20 expression of different tumor types and normal tissue in TCGA were analyzed by the TIMER online database; **(B, C)** Univariate survival analysis was used to analyze the relationship between USP20 expression and survival time in Pan cancer; **(B)** forest plot showing the relationship between USP20 expression and OS; **(C)** KM curves of high and low USP20 expression in Pan cancer significantly associated with OS survival; (*P < 0.05; **P < 0.01; ***P < 0.001; ****P < 0.0001; NS, not significant).

We investigated the expression of USP20 in pan-cancer by applying the TIMER online tool to obtain RNA-seq data in TCGA. We discovered that USP20 expression levels were increased in cholangiocarcinoma (CHOL), colon adenocarcinoma (COAD), esophageal carcinoma (ESCA), head and neck squamous cell carcinoma (HNSC), liver hepatocellular carcinoma (LIHC), lung adenocarcinoma (LUAD), pheochromocytoma and paraganglioma (PCPG), rectum adenocarcinoma (READ), and stomach adenocarcinoma (STAD), but decreased in bladder urothelial carcinoma (BLCA), glioblastoma multiforme (GBM), kidney renal clear cell carcinoma (KIRC), thyroid carcinoma (THCA) and uterine corpus endometrial carcinoma (UCEC) compared with normal tissue (**Figure 1A**). We further performed survival analysis in pan cancer. Kaplan–Meier survival plots showed that high USP20 expression in CRC was associated with markedly shorter OS (**Figures 1B, C**). In contrast, high USP20 expression may be associated with longer OS in GBM, pancreatic adenocarcinoma (PAAD), and thymoma (THYM) (**Figures 1B, C**).

### Bioinformatics analysis of the relationship between USP20 expression and CRC patient clinicopathological characteristics

**Figure 2:**
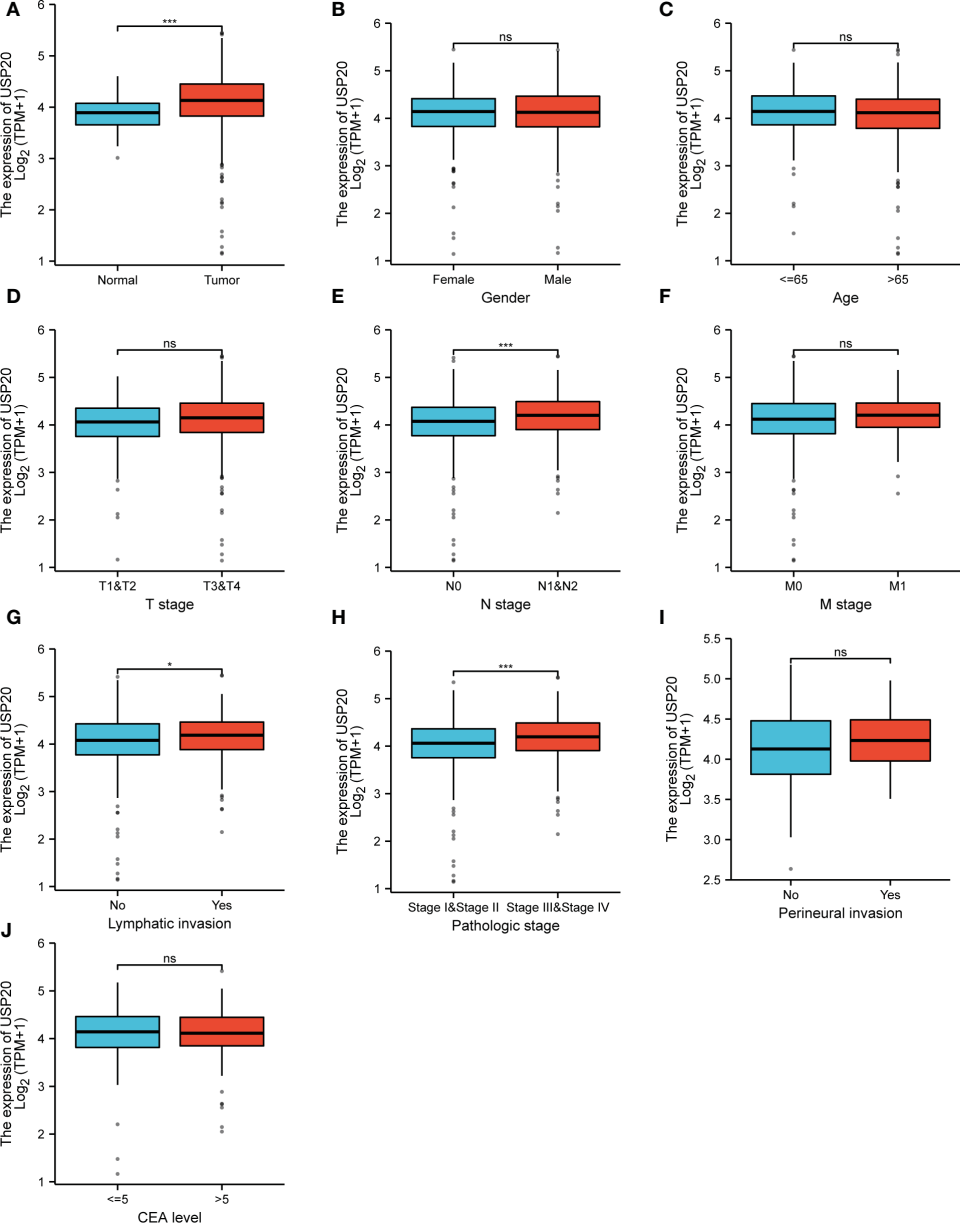
Relationship between USP20 expression and CRC patient clinicopathological characteristics in TCGA; **(A)** analysis of USP20 expression between normal and cancer tissues; **(B)** analysis of USP20 expression between female CRC patients and male CRC patients; **(C)** analysis of USP20 expression between CRC patients who were younger than or older than 65 years of age; **(D)** analysis of USP20 expression between T1&T2 CRC patients and T3&T4 CRC patients; **(E)** analysis of USP20 expression between N0 CRC patients and N1&N2 CRC patients;**(F)** analysis of USP20 expression between M0 CRC patients and M1 CRC patients; **(G)** analysis of USP20 expression between CRC patients who with lymphatic invasion or not; **(H)** analysis of USP20 expression between pathologic stage I& stage II CRC patients and stage III& stage IV CRC patients; **(I)** analysis of USP20 expression between CRC patients who with perineural invasion or not; **(J)** analysis of USP20 expression between CRC patients who CEA levels more than or less than 5; (*P < 0.05; **P < 0.01; ***P < 0.001; ****P < 0.0001; NS, not significant). (*P <0.05; ***P < 0.001; NS, not significant).

We downloaded clinical data and gene expression data of CRC in TCGA and analyzed the relationship between USP20 expression and clinicopathological parameters in CRC patients. The parameters examined included sample type (normal/primary tumor), gender (female/male), age (≤65/ >65), T stage (T1&T2/T3&T4), N stage (N0/N1&N2), M stage (M0/M1), pathologic stage (stage I & stage II/ stage III& stage IV), lymphatic invasion (no/yes), perineural invasion (no/yes), and CEA level (≤5/ >5). USP20 expression was higher in CRC tissues than in normal samples (**Figure 2A**). No correlations between USP20 expression and age, gender, T stage were found (**Figures 2B–D**). USP20 expression was significantly higher in CRC in N1&N2 stages than N0 stage (**Figure 2E**) and no correlations between USP20 expression and M stage was found (**Figure 2F**). Furthermore, USP20 expression was significantly higher in CRC with lymphatic invasion than CRC without lymphatic invasion (**Figure 2G**). These results suggest a potential function for USP20 in lymph node metastasis of CRC. USP20 expression was markedly higher in advanced pathologic stage (III& IV) CRC samples than in early pathologic stage (I& II) CRC samples (**Figure 2H**) USP20 expression is not significantly correlated with perineural invasion and CEA level, indicating that USP20 expression correlates with CRC progression.

### Validation of USP20 expression in CRC

**Figure 3:**
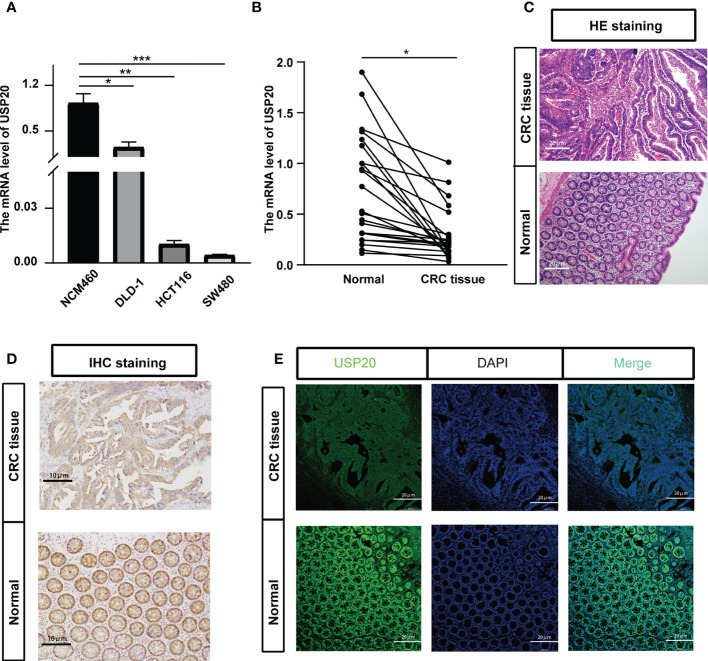
Verification the USP20 expression in CRC samples; **(A)** Quantitative real-time PCR (qRT-PCR) was performed to detect the mRNA level of USP20 in CRC cell lines and normal human colonic epithelial (NCM460) cells; **(B)** Quantitative real-time PCR (qRT-PCR) was performed to detect the mRNA level of USP20 in 23 CRC tissues and correspond adjacent normal tissues; **(C)** HE staining clarified the CRC tissue and normal tissue in the pathological tissue(100x); **(D)** Immunohistochemical (IHC) staining was used to detect the difference in expression of USP20 between CRC tissues and adjacent normal tissues(200x); **(E)** immunofluorescence assays was used to determine the subcellular localization of the USP20 protein in CRC tissues(200x); (*P < 0.05; **P < 0.01; ***P < 0.001; ****P < 0.0001; NS, not significant). (*P <0.05; **P < 0.01; ***P < 0.001).

TCGA data showed that USP20 was highly expressed in CRC compared with normal tissue; however, the opposite result was seen in the GEO data analysis (**Supplement Figure 1**). We therefore examined the expression of USP20 in CRC and normal samples in cell lines and tissues. We performed qRT-PCR of USP20 mRNA in CRC cell lines and NCM460 cells. The results showed that USP20 mRNA levels were lower in CRC cell lines compared with NCM460 cells (**Figure 3A**). We further examined USP20 mRNA in 23 pairs of CRC and adjacent normal tissue specimens and found that USP20 was expressed at low levels in CRC tissues compared with normal adjacent tissues (**Figure 3B**). We then examined USP20 protein expression in 10 pairs of CRC and adjacent normal tissues by immunohistochemistry. HE staining was performed to distinguish CRC tissue and normal tissue (**Figure 3C**). Scoring revealed that the protein expression of USP20 was down-regulated in CRC tissues compared with the cancer adjacent tissues (**Figure 3D**). We additionally analyzed the expression and subcellular distribution of USP20 by immunofluorescence. The results showed that USP20 expression was lower in CRC tissue than that in normal adjacent tissues; furthermore, USP20 was mainly located in the cell cytoplasm (**Figure 3E**).

### Association between USP20 levels and clinicopathological characteristics

**Figure 4:**
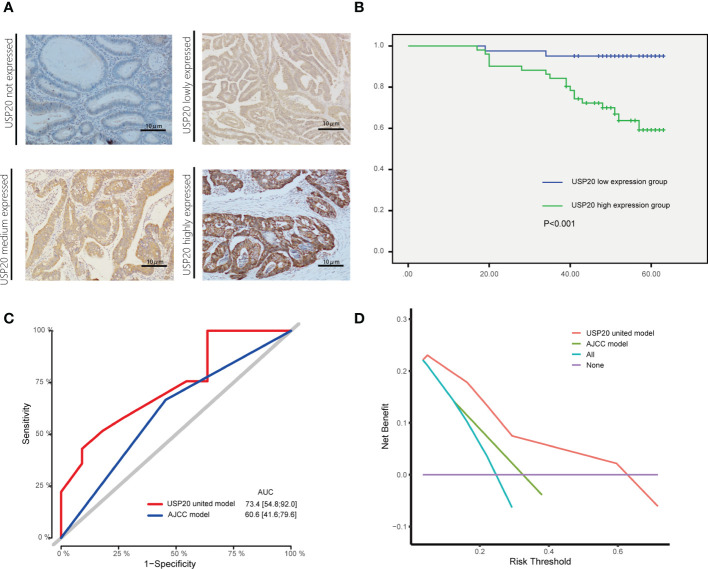
Association between USP20 expression and postoperative survival in IHC cohort CRC patients; **(A)** The USP20 IHC samples were classified into not expressed, lowly expressed, medium expressed and highly expressed categories; **(B)** The Kaplan-Meier(KM) method was used to compare the relationship between different USP20 expression levels and the overall survival of patients; **(C)** The receiver operating characteristic (ROC) curve analysis was used to evaluate the prognostic validity of the USP20 joint model and AJCC model; **(D)** The decision curve analysis(DCA) was used to estimate the clinical usefulness and net benefit of USP20 joint model and AJCC model.

Next, the relationship between USP20 expression levels and clinicopathological characteristics in CRC patients was examined. The tumor specimens from 92 patients examined by immunohistochemistry were scored according to the intensity and extent of staining (**Figure 4A**). The 92 patients were divided into USP20-high (n=51) and USP20-low (n=41) groups using the median USP20 expression levels in this cohort. High USP20 expression was shown to be associated with lymph node metastasis (P<0.001) and American Joint Committee on Cancer (AJCC) stage (P<0.001, **Table 1**). There was no significant relationship between USP20 expression levels and other clinical characteristics including gender, age, tumor location, tumor differentiation grade, and adjuvant chemotherapy status. Our results suggest that high USP20 expression is closely associated with lymph node metastasis in CRC patients.

### Association between USP20 expression and postoperative survival

The relationship between USP20 protein expression and OS was analyzed in 92 CRC patients in the immunohistochemical cohort. KM curves showed that the OS of the USP20 high expression group was significantly shorter than the OS of the USP20 low expression group (**Figure 4B**). Univariable and multivariable Cox regression analyses were performed to determine predictive factors for OS. The results revealed that USP20 expression was an independent risk factor for CRC prognosis (**Table 3**). We then constructed a base risk prediction multivariable Cox regression model with USP20 expression, perineural invasion, adjuvant chemotherapy and AJCC stage. We assessed the performance of the model by ROC curve analysis. The results indicated that the USP20 joint model performed better than the AJCC stage model in predicting 5-year survival (**Figure 4C**). We subsequently compared the clinical performance of the USP20 joint model to the AJCC stage model using decision curve analysis. The result showed that the USP20 joint model was associated with a higher net benefit than the AJCC stage model (**Figure 4D**). These results suggest that the USP20 joint model outperforms the AJCC stage model.

**Table 3:** Univariate and multivariate analyses of factors influencing patient overall survival in the first study cohort.

		Univariate analysis				Multivariate analysis			
Variable	Comparison	HR	95% CI		P-value	HR	95% CI		P-value
Sex	Female/male	0.56	0.19	1.68	0.303	–	–	–	–
Age (years)	≥60/<60	1.22	0.5	2.94	0.663	–	–	–	–
Tumor location	Rectum+left colon/right colon	1.06	0.43	2.6	0.895	–	–	–	–
Tumor size (cm)	≥5/<5	3.38	0.78	14.57	0.102	–	–	–	–
Degree of differentiation	Poor/well + moderate	2.01	0.82	4.91	0.128	–	–	–	–
perineural invasion	Postive/negative	4.28	1.75	10.45	0.001	3.78	1.23	11.65	0.02
vascular invasion	Postive/negative	2.51	1.02	6.15	0.045	0.96	0.31	2.99	0.944
AJCC stage	IIII+IV/I + II	3.61	1.31	9.96	0.013	1.47	0.47	4.57	0.506
adjuvant chemotherapy	Yes/no	0.44	0.18	1.07	0.071	0.41	0.16	1.02	0.055
USP20 protein level	High/low	8.49	1.97	36.64	0.004	6.64	1.43	30.78	0.016

HR, hazard ratio; 95% CI, 95% confidence interval; USP20, Ubiquitin Specific Peptidase 20.

### Correlation between immune infiltration and USP20 expression in CRC

**Figure 5:**
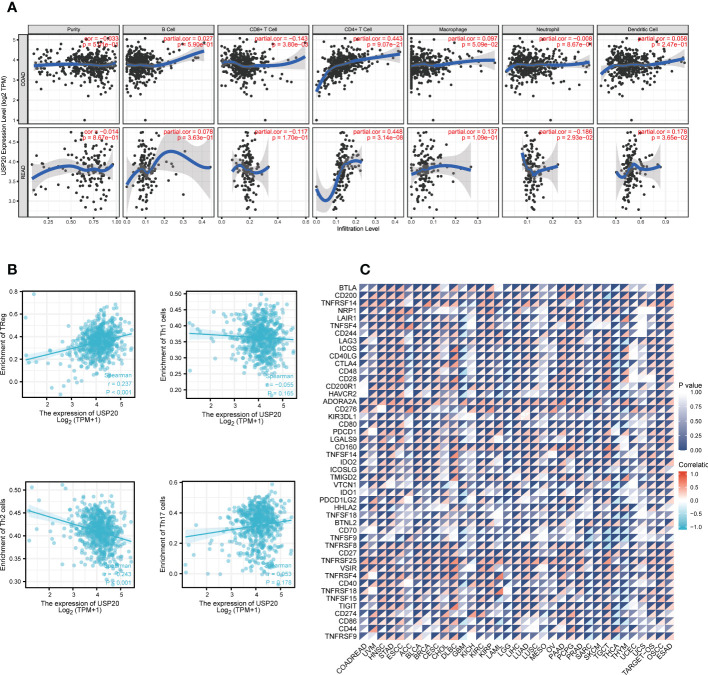
Correlation between immune and USP20 expression in CRC; **(A)** Using TIMER database to analyze the correlation between gene expression and immune cell scores **(B)** Using ssGSEA method to calculate the correlation between USP20 and CD4 + T cell subsets; **(C)** Correlation analysis of USP20 expression in pan-cancer with immune checkpoint gene expression.

Immune infiltration plays a crucial role in promoting tumor progression. TIMER was used to investigate the relationships between USP20 expression and immune cell infiltration in CRC. The results showed that the expression of USP20 was significantly positively correlated with CD4+ T cells and negatively correlated with CD8+ T cells in COAD. In READ, the expression of USP20 was significantly positively correlated with CD4+ T cells and dendritic cells and negatively correlated with neutrophils (**Figure 5A**). Considering that USP20 is closely related to CD4+ T cells in both COAD and READ, we further analyzed the subsets of CD4+ T cells in TCGA COAD&READ database through GSVA. The results showed that the expression of USP20 positively correlated with Treg cells and negatively correlated with Th2 cells (**Figure 5B**). Tumors can elude immune cytotoxicity through immune checkpoints. Therefore, we explored the relationship between USP20 expression and immune checkpoints. More than 40 common immune checkpoint genes were collected for molecular correlation analysis in pan cancer. In a variety of tumors, USP20 expression positively correlated with the expression levels of several immune checkpoint genes, including ADORA2A, CD160, CD27 and TNFRSF25 genes (**Figure 5C**). This suggests that USP20 may regulate tumor immunity by regulating the expression level of specific immune checkpoint genes. Then, we validated the gene expression correlation with 20 fresh colon cancer tissues collected in our center. The correlation between USP20 expression and immune cell marker CD4, immune checkpoint gene ADORA2A, CD160, CD200 and CD27 were analyzed by qRT-PCR. The results showed that USP20 was positively correlated with the expression of all the above genes and the correlation of USP20 with CD4 and CD200 expression was statistically significant. (**Supplement Figure 2**)

### Analysis of the correlation between USP20 expression and multidrug resistance–related genes and chemotherapeutics

**Figure 6:**
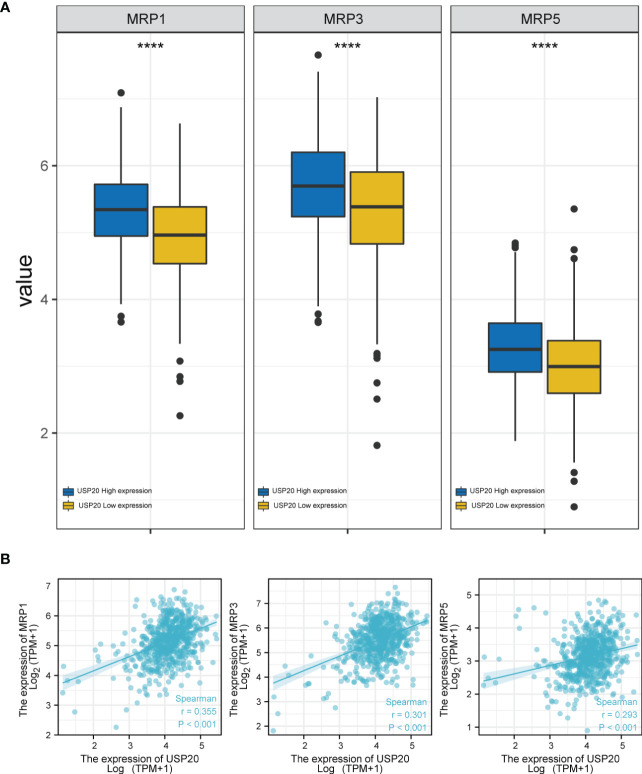
The relationship between anti-tumor drug genes and USP20 expression group; **(A)** Expression of anti-tumor drug genes (MRP1, MRP3, MRP5) in different USP20 expression CRC group; **(B)** The co-expression analysis between USP20 and anti-tumor drug genes (MRP1, MRP3, MRP5) in CRC; (*P < 0.05; **P < 0.01; ***P < 0.001; ****P < 0.0001; NS, not significant).

**Figure 7:**
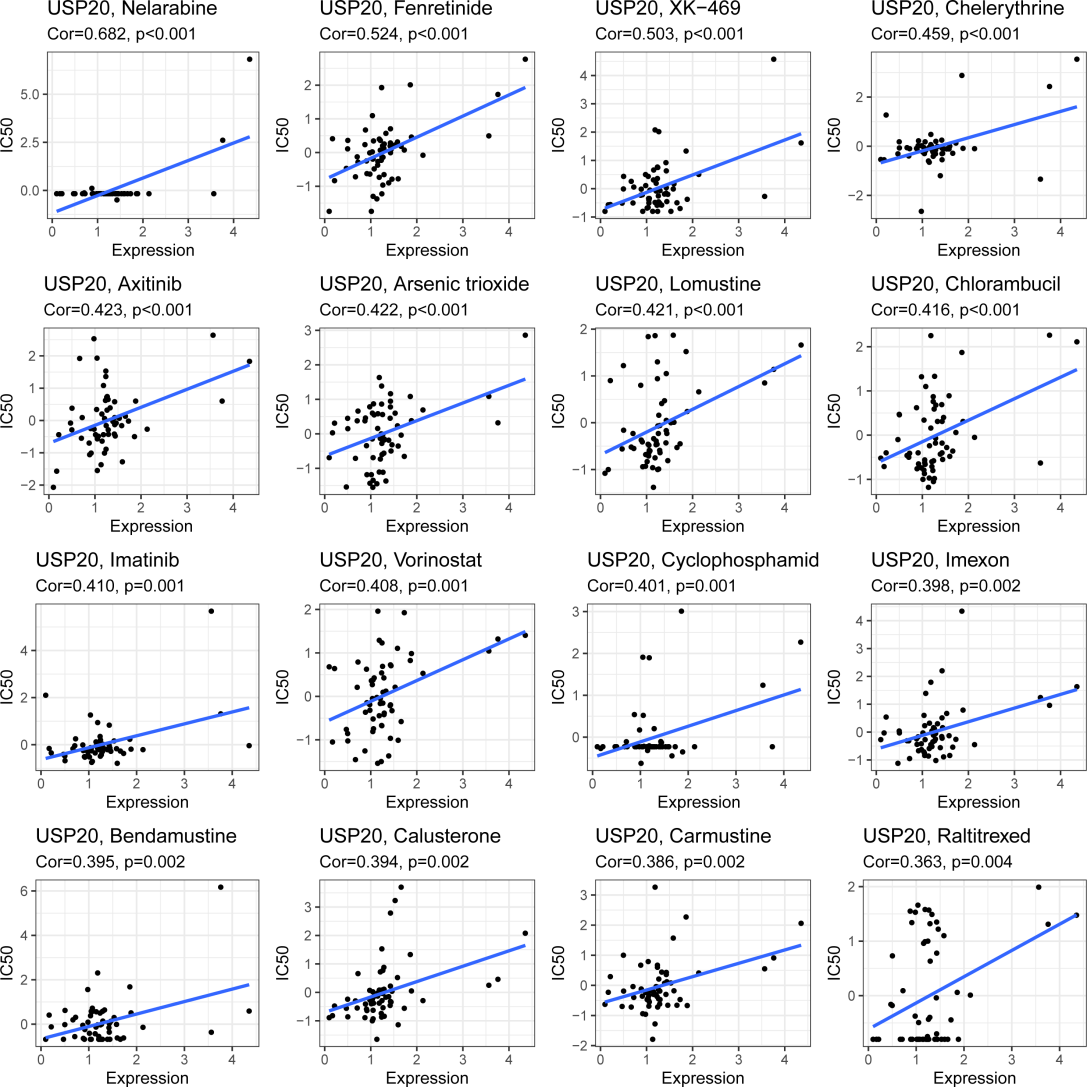
Scatter plots of the association between the USP20 expression and anti-tumor drugs IC50.

To assess the role of USP20 in predicting resistance to CRC chemotherapy, we analyzed the expression levels of multidrug resistance–associated genes in different USP20 expression groups and the correlation between USP20 expression and drug resistance–associated gene expression. The results showed that drug resistance genes, such as MRP1, MRP3 and MRP5 genes, were more highly expressed in the USP20 high expression group (**Figure 6A**). Correlation analysis showed that USP20 expression positively correlated with the expression of MRP1, MRP3 and MRP5 genes (**Figure 6B**), suggesting that USP20 expression may be associated with drug resistance in CRC. To further explore the relationship between USP20 and chemoresistance, we analyzed USP20 expression and the IC50 of chemotherapeutic drugs in tumor cells. The results showed that USP20 negatively correlated with the sensitivity of many chemotherapeutic drugs (**Figure 7**). Among these drugs, lomustine and raltitrexed are used in the clinical treatment of CRC, and raltitrexed is the main drug used in the treatment of advanced rectal cancer. Together these findings indicate that USP20 expression has the potential to predict chemotherapy resistance in CRC and may also be an intervention target for chemotherapy resistance in CRC.

### USP20 promotes the metastasis of CRC cells

**Figure 8:**
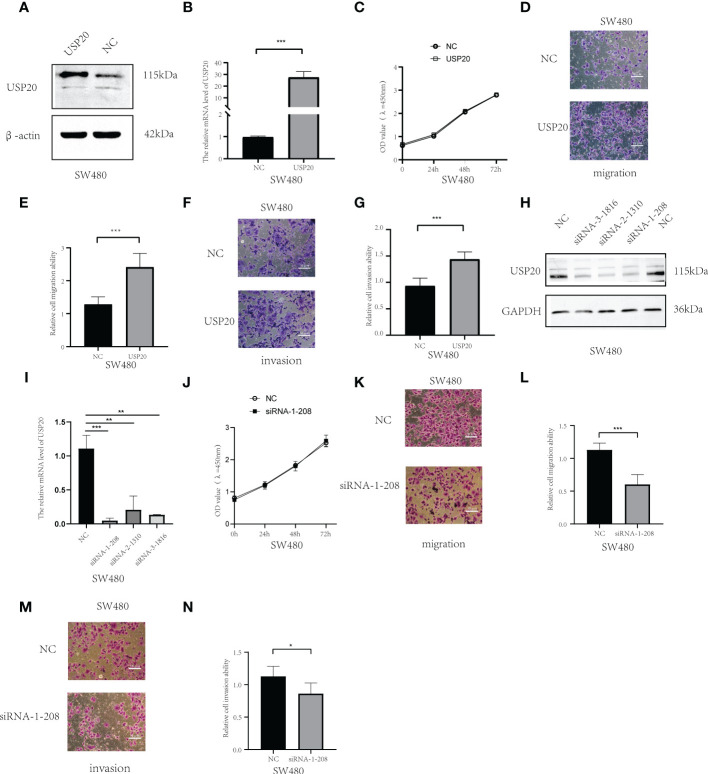
USP20 promotes the metastasis of CRC cells; **(A)** USP20 Plasmid transfection efficiency was determined by Western blots. **(B)**; USP20 Plasmid transfection efficiency was determined by qRT-PCR. **(C)** The CCK-8 results showed that the overexpression of USP20 displayed no obvious effects in cell proliferation of SW480. **(D–G)** Overexpression of USP20 enhanced CRC cell migration and invasion abilities were demonstrated *via* Transwell migration assays and Transwell invasion assays. **(H)** Western blots showing knockdown efficiency of the siRNAs. **(I)** The efficiency of siRNAs was verified by qRT-PCR. **(J)** The CCK-8 results showed that the knockdown of USP20 displayed no obvious effects in cell proliferation of SW480. **(K–N)** Knowdown of USP20 decreased CRC cell migration and invasion abilities were demonstrated *via* Transwell migration assays and Transwell invasion assays. (*P < 0.05; **P < 0.01; ***P < 0.001; NS, not significant).

To examined whether USP20 promotes CRC progression, the cells were transiently transfected with USP20 plasmid or negative plasmid. We established SW480 cells that overexpressed USP20 or negative control by plasmid transfection. qRT-PCR and Western Blot analysis confirmed that USP20 level was markedly upregulated in the cells transfected with USP20-expressing plasmid (**Figures 8A, B**). We next examined the effect of USP20 on CRC cell proliferation using CCK-8 assay. The results showed that overexpression of USP20 had no effects on the cell proliferation of SW480 cells (**Figure 8C**). We next used a Transwell chamber to assess the effects of USP20 on migration and invasiveness. The results showed that overexpression of USP20 significantly enhanced SW480 cell migration and invasion (**Figures 8D–G**). Then, we knocked down USP20 in SW480 cells using siRNAs. qRT-PCR and Western blots confirmed that USP20 level was markedly downregulated in the cells transfected with the siRNAs (**Figures 8H, I**). The effect of siRNA-1-208 was most obvious and was used for subsequent experiments. The CCK-8 results showed that the knockdown of USP20 displayed no obvious effects on the cell proliferation of SW480 cells (**Figure 8J**). Transwell results showed that knockdown of USP20 significantly decreased SW480 cell migration and invasion (**Figures 8K–N**). Overall, these results suggest that USP20 overexpression may promote CRC metastasis to promote cancer progression in CRC.

### Differential gene expression analysis and enrichment analysis

**Figure 9:**
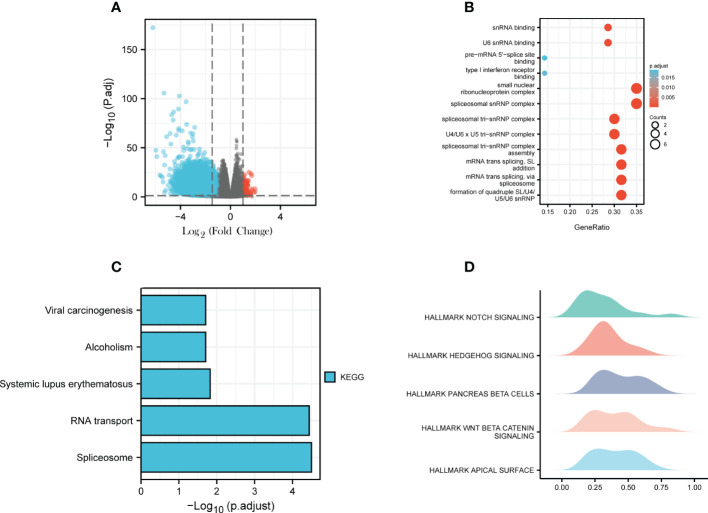
Differential Gene Expression Analysis and Enrichment Analysis; **(A)** USP20 CRC high group/ USP20 CRC low group differential expression genes analysis; volcano plot: red dots indicate significantly differentially up-regulated genes and blue dots indicate significantly differentially down-regulated genes;**(B)** GO enrichment analysis of top 100 downregulated expression genes related to USP20;**(C)** KEGG enrichment analysis of top 100 downregulated expression genes related to USP20;**(D)** GSEA analysis of USP20 in TCGA.

To explore the possible mechanism of action of USP20 in CRC, differential gene analysis and enrichment analysis were performed in TCGA CRC samples. First, differential expression analysis was performed to identify DEGs between high and low USP20 CRC sample groups in TCGA. We obtained a total of 5,413 DEGs (|log2(FC)| > 1, Padj< 0.05), including 117 upregulated genes and 5296 downregulated genes (**Figure 9A**; **Table S3**). Among the DEGs, we selected the top 100 downregulated genes for GO and KEGG enrichment analyses. GO enrichment analysis showed that the DEGs were mainly concentrated in spliceosome snRNP complex, small nuclear ribonucleoprotein complex, spliceosome tri-snRNP complex, and U4/U6 x U5 tri−snRNP complex (**Figure 9B**). KEGG analysis showed that the DEGs were enriched in spliceosome and RNA transport pathways (**Figure 9C**). We then performed GSEA for hallmark gene sets and found that the Notch pathway, Hedgehog pathway and beta-catenin pathway were enriched (**Figure 9D**).

## Discussion

CRC is one of the most common and deadliest cancers worldwide, in part as the frequency of colonoscopy in the average-risk population is low. Patients with CRC usually have no obvious symptoms in the early stage, which leads to many CRC patients presenting with advanced stage at initial diagnosis. Therefore, it is very helpful to find new non-invasive markers and new therapeutic targets to improve the diagnosis and treatment of CRC.

The identification of prognostic markers of CRC is not only helpful to evaluate the prognostic status of patients, but also help screen treatment-related target molecules. For example, RAS gene mutation is not only related to the prognosis of CRC patients (23), but it also predicts the efficacy of anti-EGFR treatment in CRC patients (24). In the study of immunotherapy, researchers found that targeting NKG2A enhances the anti-tumor CD8 T cell response in human CRC (25). Therefore, the use of bioinformatics may help identify potential prognostic markers for CRC and it is an efficient way to improve the level of diagnosis and treatment of CRC.

Deubiquitination is a reversal of the ubiquitination process and is mediated by deubiquitinating enzymes. Similar to ubiquitination, deubiquitination is also involved in many tumor-related biological processes. Researchers have confirmed that deubiquitination plays a key role in regulating T cell immune response (26). It also participates in fat metabolism and exacerbates colorectal carcinogenesis by stabilizing ME1 (27). NLRP7 deubiquitination by USP10 promotes tumor progression and tumor-associated macrophage polarization in CRC (28). These studies suggest that deubiquitination has great potential in the development of treatments for CRC. In recent years, USP20 has been found to play a crucial role in a variety of biological processes (9, 11, 29). Only one study thus far reported the role of USP20 in CRC (30). The authors confirmed that USP20 enhances invasive ability in a small number of CRC cell lines. However, the specific mechanisms and prognostic significance of USP20 expression in CRC have been unknown.

Through analysis of TCGA database, we found that USP20 was differentially expressed in a variety of cancers compared with normal tissues, suggesting that it is a tumor-associated molecule. In TCGA database, USP20 was shown to be highly expressed in CRC compared with normal tissues. However, the GEO database showed low expression of USP20 in CRC compared with normal tissues. In this study, we found that USP20 expression in CRC was lower than that in normal tissues through analysis of 22 pairs of CRC specimens, 10 pairs of immunohistochemical specimens, and cell lines, suggesting that USP20 is a CRC-related differentially expressed gene and is expressed at low levels in CRC.

We further explored the relationship between USP20 expression and the survival prognosis of CRC patients. CRC patients with high expression of USP20 were shown to have a shorter survival compared with those with low expression in both TCGA database and our cohort patients. These results suggest USP20 may predict the survival prognosis of patients with CRC. Additional analyses in TCGA samples and our cohort showed that USP20 was associated with lymph node metastasis in patients with CRC. We speculate that USP20 may promote tumor progression by promoting lymph node metastasis. In cell line experiments, we found that USP20 overexpression enhanced the migration and invasive ability of CRC cells, which is consistent with the findings of the clinical correlation analysis.

Furthermore, we identified USP20 high expression as an independent risk factor for poor prognosis in CRC patients by univariate and multivariate Cox regression analysis. Using the Cox multivariate regression results, we constructed a predictive model for the prognosis of CRC. Through ROC and DCA analysis, we found that the predictive efficiency and net benefit of the newly constructed model was higher than that of the conventional TNM model. Our work provides a new model for further improving the prognosis of patients with CRC.

Tumors and the immune system are closely related. The immune system functions to exert killing effects on tumors and can inhibit the progression of tumors. However, a strong anti-tumor immune response will trigger a physiological response, which aims at inhibiting effector T cells, preventing tissue damage and maintaining tissue stability. These physiological reactions protect and even promote tumors. A variety of inhibitory pathways are known to play a role in the tumor microenvironment, including cells such as Th2 macrophages and immature T regulatory cells (Tregs), and molecules such as checkpoints that control T cell differentiation (such as CTLA-4 and IDO) and effector function (such as PD-1). We found that USP20 expression was significantly positively correlated with Treg cells in CRC tissue. Tregs are immune inhibitory lymphocytes that often accumulate within the tumor microenvironment and are regulated by tumor cells through cytokines/chemokines (31, 32). Tregs promote CRC progression by inhibiting the antitumor activity promoted by natural killer cells and CD8 T cells (33). Our results suggest that USP20 may be involved in regulating the immune infiltration of Treg cells in CRC tissues, so as to promote the progression of CRC. Immune checkpoint inhibitors are a hot spot in tumor therapy. Molecular correlation analysis showed that USP20 significantly and positively correlated with the expression of multiple immune checkpoints such as ADORA2A and CD160 in a variety of cancers. This suggests that USP20 may affect the tumor immune response by regulating the expression of immune checkpoints. Studies on immune checkpoints have shown that ADORA2A is a main pathway for Treg cells to inhibit CD8+ T cell viability (34), which is consistent with our results in the above immune infiltration studies.

Chemotherapy resistance is one of the major challenges in current oncology treatment. In the treatment of CRC, pharmacological chemotherapy is the main treatment for advanced CRC. Chemotherapy can reduce the recurrence of CRC after surgery. This study showed that USP20 expression in CRC positively correlated with multi-drug resistant gene expression. USP20 expression also correlated with drug resistance to various anticancer drugs, suggesting that USP20 expression may have a role in predicting drug resistance in CRC patients. This finding also suggests that USP20 may be involved in the mechanism of drug resistance in CRC.

Through differential and enrichment analysis, we explore the possible mechanisms of USP20 in CRC. We conducted single-gene level differential expression analysis and identified 5413 DEGs, including 117 upregulated and 5296 downregulated genes. This result suggests that USP20 may play a role in CRC by reducing the expression of related genes. Enrichment analysis of the top 100 significantly downregulated genes showed that the DEGs were mainly enriched in nucleic acid modifications and transport. GSEA hallmark analysis of DEGs showed the DEGs were mainly enriched in the Notch pathway, Hedgehog pathway and beta-catenin pathway. Previous studies have shown that the above pathways are closely related to tumor cell migration, invasion, and chemoresistance (35–39). We therefore hypothesize that USP20 regulates the NOTCH pathway, HEDGEHOG pathway, BETA CATENIN pathway through affecting mRNA modification and transport, thereby promoting metastasis and chemoresistance in colorectal cancer.

In summary, USP20 is downregulated in CRC and associated with the prognosis of CRC. USP20 may promote tumor metastasis and is associated with immune infiltration and drug resistance in CRC. USP20 may act through pathways such as the Notch pathway, Hedgehog pathway and beta-catenin pathways. We constructed a new prognostic model related to USP20, which provides a new option to further improve the prognosis prediction of patients with CRC.

## Data availability statement

The datasets presented in this study can be found in online repositories. The names of the repository/repositories and accession number(s) can be found in the article/**Supplementary Material**.

## Ethics statement

The studies involving human participants were reviewed and approved by Institutional Ethics Committee of Jiangxi Cancer Hospital. The patients/participants provided their written informed consent to participate in this study.

## Author contributions

YC designed the study. CZ performed graphing and writing. RJ and ZL performed immunohistochemistry experiments. JL and PW performed cytology experiments. QT and RJ were responsible for language revisions. YC and CZ helped modify articles and supervise the study. authors contributed to the article, reviewed the manuscript, and approved the submitted version. XC and LJ help completed the experiments required for article revision. All authors contributed to the article and approved the submitted version.

## Supplementary material

Supplementary Figure 1 The USP20 expression in CRC from GEO database (GSE32323);

Supplementary Figure 2 Gene co-expression correlation analysis of USP20 in colorectal cancer. Twenty cases of fresh colorectal cancer tissues were collected for gene co-expression analysis. **(A)**. Correlation analysis of USP20 with CD4 T-cell markers. **(B)**. Correlation analysis of USP20 with the immune checkpoint gene ADORA2A. **(C)**. Correlation analysis of USP20 with the immune checkpoint gene CD160. **(D)**. Correlation analysis of USP20 with the immune checkpoint gene CD200. **(E)**. Correlation analysis of USP20 with the immune checkpoint gene CD27. Correlation was examined by Pearson’s correlation analysis. (*P < 0.05; **P < 0.01; ***P < 0.001; ****P < 0.0001; NS, not significant)
