# Supplementary material for: USP20 is a predictor of poor prognosis in colorectal cancer and associated with lymph node metastasis, immune infiltration and chemotherapy resistance
Source: Front Oncol. 2023 Feb 16;13:1023292. doi: 10.3389/fonc.2023.1023292 (PMC9978104; doi:10.3389/fonc.2023.1023292)
Supplement: Supplementary Figure 1 — The USP20 expression in CRC from GEO database (GSE32323); [file DataSheet_1.zip › Supplementary Material/Data Sheet 1.DOCX]

###Survival analysis and KM curve codes

if (!requireNamespace("survminer", quietly = TRUE))

install.packages("survminer")

library(survival)

library(survminer)

# data <- lung

# colnames(data)[5] <- "variable"

fit <- survfit(Surv(time, status) ~ variable, data = data)

print(fit)

survdiff(Surv(time, status) ~ variable, data = data)

ggsurvplot(fit = fit, data = data, pval = T)

#forest plot codes

library(ggplot2)

library(patchwork)

dat_ci

# Characteristics y.pos hr low.ci high.ci

# 1 T stage 9 NA NA NA

# 2 T1&T2 8 2.448 1.523 3.936

# 3 T3 7 2.862 1.496 5.475

# 4 N stage 6 NA NA NA

# 5 N0 5 2.570 1.621 4.074

# 6 N1 4 1.570 0.621 2.074

# 7 M stage 3 NA NA NA

# 8 M0 2 2.590 1.629 4.118

# 9 M1 1 1.548 1.123 2.536

p2 <- ggplot() +

geom_errorbar(data = dat_ci, aes(y = y.pos, xmin = low.ci, xmax = high.ci), width = 0.25) +

geom_point(data = dat_ci, aes(y = y.pos, x = hr), colour = "#0066DD") +

geom_line(aes(x = c(1, 1), y = c(-Inf, 9.5)), linetype = 2, size = 0.5) +

geom_line(aes(x = c(-Inf,Inf), y = c(9.5, 9.5))) +

geom_line(aes(x = c(-Inf,Inf), y = c(10.5, 10.5))) +

scale_y_continuous(limits = c(0.5,10.5)) +

theme_void() +

theme(axis.text.x = element_text(), axis.line.x = element_line(),

axis.ticks.length.x = unit(0.1, "cm"), axis.ticks.x = element_line())

dat_text

# label_name y.pos

# 1 Characteristics 10

# 11 T stage 9

# 2 T1&T2 8

# 3 T3 7

# 4 N stage 6

# 5 N0 5

# 6 N1 4

# 7 M stage 3

# 8 M0 2

# 9 M1 1

p1 <- ggplot() +

geom_text(data = dat_text, aes(x = 1, y = y.pos, label = label_name), size = 2.5) +

geom_line(aes(x = c(-Inf,Inf), y = c(10.5, 10.5))) +

geom_line(aes(x = c(-Inf,Inf), y = c(9.5, 9.5))) +

scale_y_continuous(limits = c(0.5,10.5)) +

theme_void() +

theme(axis.line.x = element_line())

#TCGA Expression data download

library(TCGAbiolinks)

library(dplyr)

library(DT)

getGDCprojects()$project_id

TCGAbiolinks:::getProjectSummary("TCGA-LIHC")#Use LIHC as an example

query <- GDCquery(project = "TCGA-LIHC", data.category = "Transcriptome Profiling", data.type = "Gene Expression Quantification", workflow.type = "HTSeq - FPKM"

GDCdownload(query, method = "api", directory = ".//1.data")

mRNAmatrix<- GDCprepare(query,directory = ".//1.data")

library(SummarizedExperiment)

mRNAmatrix <- assay(mRNAmatrix)

mRNAmatrix <- as.data.frame(mRNAmatrix)

save(mRNAmatrix, file = "LIHC.rda"

library(tidyverse)

library(ggplot2)

library(reshape2)

library(car)

library(rstatix)

set.seed(100)

data <- data.frame(x = rnorm(100, 2, 1), y = rnorm(100, 1, 1))

data2 <- melt(data)

data3 <- lapply(data, function(x) get_summary_stats(data.frame(x)))

data3

# $x

# # A tibble: 1 x 13

# variable n min max median q1 q3 iqr mad mean sd se ci

# <chr> <dbl> <dbl> <dbl> <dbl> <dbl> <dbl> <dbl> <dbl> <dbl> <dbl> <dbl> <dbl>

# 1 x 100 -0.272 4.58 1.94 1.39 2.66 1.26 0.974 2.00 1.02 0.102 0.203

#

# $y

# # A tibble: 1 x 13

# variable n min max median q1 q3 iqr mad mean sd se ci

# <chr> <dbl> <dbl> <dbl> <dbl> <dbl> <dbl> <dbl> <dbl> <dbl> <dbl> <dbl> <dbl>

# 1 x 100 -1.14 3.17 0.927 0.568 1.45 0.878 0.648 1.01 0.796 0.08 0.158

data3 <- rbind(data3[[1]], data3[[2]])

data3[1] <- c("x", "y")

## Shapiro-Wilk normality test

lapply(data, function(x) shapiro.test(x))

# $x

#

# Shapiro-Wilk normality test

#

# data: x

# W = 0.98836, p-value = 0.535

#

#

# $y

#

# Shapiro-Wilk normality test

#

# data: x

# W = 0.98532, p-value = 0.3348

## Levene's Test

leveneTest(value~variable, data = data2)

# Levene's Test for Homogeneity of Variance (center = median)

# Df F value Pr(>F)

# group 1 4.4476 0.03621 *

# 198

# ---

# Signif. codes: 0 ‘***’ 0.001 ‘**’ 0.01 ‘*’ 0.05 ‘.’ 0.1 ‘ ’ 1

t.test(value~variable, data = data2, var.equal = T)

# Two Sample t-test

#

# data: value by variable

# t = 7.6613, df = 198, p-value = 8.012e-13

# alternative hypothesis: true difference in means is not equal to 0

# 95 percent confidence interval:

# 0.7364913 1.2470521

# sample estimates:

# mean in group x mean in group y

# 2.002913 1.011141

t.test(value~variable, data = data2, var.equal = F)

# Welch Two Sample t-test

#

# data: value by variable

# t = 7.6613, df = 186.92, p-value = 9.657e-13

# alternative hypothesis: true difference in means is not equal to 0

# 95 percent confidence interval:

# 0.7363983 1.2471452

# sample estimates:

# mean in group x mean in group y

# 2.002913 1.011141

wilcox.test(value~variable, data = data2)

# Wilcoxon rank sum test with continuity correction

#

# data: value by variable

# W = 7844, p-value = 3.711e-12

# alternative hypothesis: true location shift is not equal to 0

summary(aov(value~variable, data = data2))

# Df Sum Sq Mean Sq F value Pr(>F)

# variable 1 49.18 49.18 58.7 8.01e-13 ***

# Residuals 198 165.90 0.84

# ---

# Signif. codes: 0 ‘***’ 0.001 ‘**’ 0.01 ‘*’ 0.05 ‘.’ 0.1 ‘ ’ 1

ggplot(data2, aes(x = variable, y = value, color = variable, fill = variable)) +

geom_violin(alpha = 0.2) +

theme_bw()

ggplot(data2, aes(x = variable, y = value, color = variable, fill = variable)) +

geom_violin(alpha = 0.2) +

geom_point(position = position_jitter(0.3)) +

theme_bw()

ggplot(data2, aes(x = variable, y = value, color = variable, fill = variable)) +

geom_boxplot(alpha = 0.2) +

geom_point(position = position_jitter(0.3)) +

theme_bw()

ggplot(data2, aes(x = variable, y = value, color = variable, fill = variable)) +

geom_violin(alpha = 0.1) +

geom_boxplot(alpha = 0.1) +

geom_point(position = position_jitter(0.3)) +

theme_bw()

ggplot() +

geom_violin(data = data2, aes(x = variable, y = value, color = variable, fill = variable), alpha = 0.1) +

geom_errorbar(data = data3, aes(x = variable, ymin=mean-sd, ymax=mean+sd), width = 0.2)

#COX

#1.载入包

library(survival)

library(plyr)

#2.清理工作环境

rm(list = ls())

#3.读入数据

aa<- read.csv('USP20生存分析 - 副本.csv')

#4.查看数据前6行

head(aa)

#5.查看数据数据性质

str(aa)

#6.查看生存或死亡人数

aa$status<-factor(aa$status)

summary(aa$status)#结局为复发，0为复发，1未复发

names(aa)

aa <- aa[,-c(1:5,7,10,17:18,21)]

table(aa$部位)

aa$分化 <- factor(aa$分化,levels=c('中分化','低分化'))

aa$病变最大直径 <- factor(aa$病变最大直径,levels=c('小于5cm','大于5cm'))

aa$神经侵犯 <- factor(aa$神经侵犯,levels=c('阴性','阳性'))

aa$脉管侵犯 <- factor(aa$脉管侵犯,levels=c('阴性','阳性'))

aa$部位 <- factor(aa$部位,levels=c('右半结肠','直肠+左半结肠'))

#1.构建模型的y

y<- Surv(time = aa$time,event = aa$status==1)#示例数据中，1为感兴趣事件

#2.批量单因素回归模型建立：Uni_cox_model

Uni_cox_model<-

function(x){

FML <- as.formula(paste0 ("y~",x))

cox<- coxph(FML,data=aa)

cox1<-summary(cox)

HR <- round(cox1$coefficients[,2],2)#提取HR值，保留2位小数

PValue <- round(cox1$coefficients[,5],3)#提取p值，保留3位小数

CI5 <-round(cox1$conf.int[,3],2)#提取CI，保留2位小数

CI95 <-round(cox1$conf.int[,4],2)

#将提取到的信息放入表格中（Uni_cox_model）

Uni_cox_model<- data.frame(

names <-rownames(cox1$conf.int),#第1列为亚变量名

'HR' = HR,#第2列为HR值

'CI5' = CI5,#第3列为95%ci下区间

'CI95' = CI95,#第4列为95%ci上区间

'P' = PValue)#第5列为P值

return(Uni_cox_model)#返回，开始，进行循环

}

#3-1查看原始数据变量的名字

names(aa)

#3-2输入想要进行单因素分析的变量的序号（变量所在原始数据的列数）

variable.names<- colnames(aa)[c(1,4:6,11:17)];variable.names

Uni_cox <- lapply(variable.names, Uni_cox_model)

Uni_cox<- ldply(Uni_cox,data.frame)

#将95%CI连接起来

Uni_cox$HR.CI95<-paste0(Uni_cox$HR," (",Uni_cox$CI5,'-',Uni_cox$CI95,")");Uni_cox

Uni_cox <- lapply(variable.names, Uni_cox_model)

Uni_cox<- ldply(Uni_cox,data.frame)

Uni_cox$CI<-paste0(Uni_cox$CI5,'-',Uni_cox$CI95);Uni_cox

#删除3-4列，只保留1,2,5,6列

Uni_cox<-Uni_cox[,-3:-4]

#第一列列名为'Characteristics'

colnames(Uni_cox)[1] <- 'Characteristics'

#查看结果

View(Uni_cox)

#结果保存为Excel

write.csv(Uni_cox,"单因素Cox回归三线表.csv")

#DCA

#1.载入包

library(tableone)

library(survival)

library(plyr)

library(broom)

library(coxphf)

library(rms)

library(ggDCA)

#2.清理工作环境

rm(list = ls())

#3.读入数据

aa<- read.csv('USP20生存分析 - 副本.csv')

#4.查看数据前6行

head(aa)

#5.查看数据数据性质

str(aa)

#使用cph（）函数时运行

#即报错：adjustment values not defined here or with datadist.....时

bb<-datadist(aa)

options(datadist='bb')

#6.查看生存或死亡人数

aa$status<-factor(aa$status)

summary(aa$status)#结局为复发，0为复发，1未复发

names(aa)

aa <- aa[,-c(1:5,7,10,17:18,21)]

table(aa$部位)

aa$分化 <- factor(aa$分化,levels=c('中分化','低分化'))

aa$病变最大直径 <- factor(aa$病变最大直径,levels=c('小于5cm','大于5cm'))

aa$神经侵犯 <- factor(aa$神经侵犯,levels=c('阴性','阳性'))

aa$脉管侵犯 <- factor(aa$脉管侵犯,levels=c('阴性','阳性'))

aa$部位 <- factor(aa$部位,levels=c('右半结肠','直肠+左半结肠'))

names(aa)

#1.多因素分析

model1<-coxph(Surv(time,status==1)~usp2低表达+神经侵犯+脉管侵犯+AJCC+是否化疗,

data=aa)

dca1<-dca(model1,

new.data = NULL,

times=60)

ggplot(dca1,

model.names="模型1",

linetype =F, #线型

lwd = 1.2) #线粗

model2 <-coxph(Surv(time,status==1)~AJCC,

data=aa)

dca2<- dca(model1,model2,

times=60)

#ROC

#1.载入包

library(tableone)

library(survival)

library(plyr)

library(broom)

library(coxphf)

library(rms)

#2.清理工作环境

rm(list = ls())

#3.读入数据

aa<- read.csv('USP20生存分析 - 副本 - ROC.csv')

#4.查看数据前6行

head(aa)

#5.查看数据数据性质

str(aa)

#6.查看生存或死亡人数

aa$status<-factor(aa$status)

summary(aa$status)#结局为复发，0为复发，1未复发

names(aa)

aa <- aa[,-c(1:5,7,10,17:18,21)]

table(aa$部位)

aa$分化 <- factor(aa$分化,levels=c('中分化','低分化'))

aa$病变最大直径 <- factor(aa$病变最大直径,levels=c('小于5cm','大于5cm'))

aa$神经侵犯 <- factor(aa$神经侵犯,levels=c('阴性','阳性'))

aa$脉管侵犯 <- factor(aa$脉管侵犯,levels=c('阴性','阳性'))

aa$部位 <- factor(aa$部位,levels=c('右半结肠','直肠+左半结肠'))

names(aa)

aa <- aa[,c(1:3,13,15,17)]

#1.多因素分析

f1<-coxph(Surv(time,status==1)~usp2低表达+神经侵犯+acjj+是否化疗,

data=aa,x=T)

model<- Score(list(model1=f1),

Hist(time, status==1)~0,

data = aa,

times = 60,

plots = 'roc',

metrics ="auc")

plotROC(model,

xlab="1-Specificity",

ylab="Sensitivity",

lty=1, #线型，2=虚线

cex=1.1,#字体大小

pch=2, #文字格式

lwd=2, #线粗

col="red",

legend="模型1")

f2 <- coxph(Surv(time,status==1)~acjj,

data=aa,x=T)

pk1 <- Score(list(model1 =f1,

model2 =f2),

Hist(time, status==1)~0,

data = aa,

times =60, #比较三者1年ROC

plots = 'roc',

metrics ="auc")

plotROC(pk1,

xlab="1-Specificity",

ylab="Sensitivity",

lty=1, #线型

cex=1,#字体大小

pch=2, #文字格式

lwd=2, #线粗

col=c("red","blue"),

legend=c("模型1","模型2"))

#Relevance Heat Map

library(tidyverse)

library(corrplot)

library(ggplot2)

library(ggcorrplot)

data <- read.table("~/file.txt", header = T)

rownames(data) <- data[,1]

data <- data[,-1]

corrplot(as.matrix(data))

p <- ggcorrplot(data)

#Immuno-infiltration analysis

# library(tidyverse)

library(GSVA)

library(clusterProfiler)

library(org.Hs.eg.db)

library(data.table)

library(rtracklayer)

### ssGSEA ######

## table S1 - https://doi.org/10.1016/j.immuni.2013.10.003

## pdf -> table -> read

immunity <- read.csv("~/immunity-cell-gene.csv", header = T)

# CellType AffymetrixID Symbol Gene.Symbol ENTREZ_GENE_ID

# 1 aDC 205569_at LAMP3 LAMP3 27074

# 2 aDC 207533_at CCL1 CCL1 6346

# 3 aDC 210029_at INDO IDO1 3620

# 4 aDC 218400_at OAS3 OAS3 4940

# 5 aDC 219424_at EBI3 EBI3 10148

# 6 B cells 204836_at GLDC GLDC 2731

idx <- !immunity$CellType %in% c("Blood vessels", "Normal mucosa", "SW480 cancer cells", "Lymph vessels")

immunity <- immunity[idx,]

immunity <- immunity %>%

split(., .$CellType) %>%

lapply(., function(x)(x$ENTREZ_GENE_ID))

immunity <- lapply(immunity, unique)

## Ensembl download

anno <- import('~/Homo_sapiens.GRCh38.101.gtf')

anno <- as.data.frame(anno)

anno <- anno[!duplicated(anno$gene_id),]

anno <- merge(anno, gene_symbol, by = "gene_name")

anno <- rbind(anno, data.frame(gene_name = c("KIAA1324", "IGHA1"),

gene_id = c("ENSG00000116299", "ENSG00000211895"),

ENTREZID = c("57535", "3492")))

anno <- anno[!duplicated(anno$gene_id),] ### 37417

anno <- anno[, c("gene_id", "ENTREZID")]

data <- fread("~/tpm.txt") %>%

rename("gene_id" = "V1") %>%

left_join(., anno, by = "gene_id") %>%

filter(!is.na(ENTREZID)) %>%

select(-gene_id) %>%

column_to_rownames("ENTREZID")

data <- log2(data + 1)

immu_cell <- as.data.frame(gsva(as.matrix(data), immunity, method = "ssgsea"))

### bbt plot

data <- read.table("~/file.txt", header = T)

# group aDC B cells CD8 T cells Cytotoxic cells

# 1 1.13092315 0.4709550 0.26202395 0.5944611 0.5130117

# 3 0.55644003 0.1800251 -0.07081909 0.5197230 0.2135559

# 4 0.44696904 0.3350859 0.05579749 0.5908900 0.3135561

# 5 0.05474605 0.1191767 0.02578815 0.5541712 0.2068595

# 7 0.61364297 0.1563856 0.09869185 0.5518254 0.2321028

# 8 0.41079217 0.4588979 0.50105493 0.5996277 0.4866508

data1 <- NULL

for(i in 2:25){

cor <- cor.test(data[,i], data[,1], method = "pearson")

data1 <- rbind(data1,

data.frame("group" = "a",

"cell" = colnames(data)[i],

"cor" = cor$estimate,

"p" = cor$p.value))

}

data1 <- data1[order(data1$cor),]

data1$cell <- factor(data1$cell, levels = data1$cell)

ggplot(data1, aes(x = cell, y = cor)) +

geom_segment(aes(xend=cell,yend=0)) +

geom_hline(yintercept = 0) +

geom_point(aes(col=p, fill = p, size=abs(cor))) +

coord_flip()

rm(list = ls())

options(stringsAsFactors = FALSE)

#1. 药物数据的准备

##1.1 读取药物相关数据

library(readxl)

rt1 <- read_excel(path = "DTP_NCI60_ZSCORE.xls", skip = 7)

colnames(rt1) <- rt1[1,]

rt1 <- rt1[-1,-c(67,68)]

##1.2 筛选药物标准

table(rt1$`FDA status`) #查看药物FDA status

rt1 <- rt1[rt1$`FDA status` %in% c("FDA approved", "Clinical trial"),]

rt1 <- rt1[,-c(1, 3:6)]

write.table(rt1, file = "drug.txt",sep = "\t",row.names = F,quote = F)

#2. 基因表达数据的准备

rt2 <- read_excel(path = "RNA__RNA_seq_composite_expression.xls", skip = 9)

colnames(rt2) <- rt2[1,]

rt2 <- rt2[-1,-c(2:6)]

write.table(rt2, file = "geneExp.txt",sep = "\t",row.names = F,quote = F)

#3. 药物敏感性分析

rm(list = ls())

##3.1 引用包

library(impute)

library(limma)

##3.2 读取药物输入文件

rt <- read.table("drug.txt",sep="\t",header=T,check.names=F)

rt <- as.matrix(rt)

rownames(rt) <- rt[,1]

drug <- rt[,2:ncol(rt)]

dimnames <- list(rownames(drug),colnames(drug))

data <- matrix(as.numeric(as.matrix(drug)),nrow=nrow(drug),dimnames=dimnames)

###对药物数据补缺

mat <- impute.knn(data)

drug <- mat$data

drug <- avereps(drug)

##3.3 读取表达输入文件

exp <- read.table("geneExp.txt", sep="\t", header=T, row.names = 1, check.names=F)

dim(exp)

exp[1:4, 1:4]

#3.4 提取特定基因表达

gene <- read.table("gene.txt",sep="\t",header=F,check.names=F)

genelist <- as.vector(gene[,1])

genelist

genelist <- gsub(" ","",genelist)

genelist <- intersect(genelist,row.names(exp))

exp <- exp[genelist,]

##3.5 药物敏感性计算

outTab <- data.frame()

for(Gene in row.names(exp)){

x <- as.numeric(exp[Gene,])

#对药物循环

for(Drug in row.names(drug)){

y <- as.numeric(drug[Drug,])

corT <- cor.test(x,y,method="pearson")

cor <- corT$estimate

pvalue <- corT$p.value

if(pvalue < 0.01){

outVector <- cbind(Gene,Drug,cor,pvalue)

outTab <- rbind(outTab,outVector)

}

}

}

#输出相关性分析结果

outTab <- outTab[order(as.numeric(as.vector(outTab$pvalue))),]

write.table(outTab, file="drugCor.txt", sep="\t", row.names=F, quote=F)

##3.6 可视化

library(ggplot2)

library(ggpubr)

###可视化一

plotList_1 <- list()

corPlotNum <- 16

if(nrow(outTab)<corPlotNum){

corPlotNum=nrow(outTab)

}

for(i in 1:corPlotNum){

Gene <- outTab[i,1]

Drug <- outTab[i,2]

x <- as.numeric(exp[Gene,])

y <- as.numeric(drug[Drug,])

cor <- sprintf("%.03f",as.numeric(outTab[i,3]))

pvalue=0

if(as.numeric(outTab[i,4])<0.001){

pvalue="p<0.001"

}else{

pvalue=paste0("p=",sprintf("%.03f",as.numeric(outTab[i,4])))

}

df1 <- as.data.frame(cbind(x,y))

p1=ggplot(data = df1, aes(x = x, y = y))+

geom_point(size=1)+

stat_smooth(method="lm",se=FALSE, formula=y~x)+

labs(x="Expression",y="IC50",title = paste0(Gene,", ",Drug),subtitle = paste0("Cor=",cor,", ",pvalue))+

theme(axis.ticks = element_blank(), axis.text.y = element_blank(),axis.text.x = element_blank())+

theme_bw()

plotList_1[[i]]=p1

}

###可视化二

plotList_2 <- list()

corPlotNum <- 16

if(nrow(outTab)<corPlotNum){

corPlotNum=nrow(outTab)

}

for(i in 1:corPlotNum){

Gene <- outTab[i,1]

Drug <- outTab[i,2]

x <- as.numeric(exp[Gene,])

y <- as.numeric(drug[Drug,])

df1 <- as.data.frame(cbind(x,y))

colnames(df1)[2] <- "IC50"

df1$group <- ifelse(df1$x > median(df1$x), "high", "low")

compaired <- list(c("low", "high"))

p1 <- ggboxplot(df1,

x = "group", y = "IC50",

fill = "group", palette = c("#00AFBB", "#E7B800"),

add = "jitter", size = 0.5,

xlab = paste0("The_expression_of_", Gene),

ylab = paste0("IC50_of_", Drug)) +

stat_compare_means(comparisons = compaired,

method = "wilcox.test", #设置统计方法

symnum.args=list(cutpoints = c(0, 0.001, 0.01, 0.05, 1),

symbols = c("***", "**", "*", "ns")))

plotList_2[[i]]=p1

}

#保存输出的图片

nrow <- ceiling(sqrt(corPlotNum))

ncol <- ceiling(corPlotNum/nrow)

ggarrange(plotlist=plotList_1,nrow=nrow,ncol=ncol)

ggarrange(plotlist=plotList_2,nrow=nrow,ncol=ncol)

#DEGs analysis

library(tidyverse）

library(DESeq2)

counts_mrna <- LIHC_matrix_2 %>%

column_to_rownames("gene_name")

coldata <- data.frame(c(rep("normal", 50), rep("tumor", 374)))

row.names(coldata) <- colnames(counts_mrna)

colnames(coldata) <- "condition" head(coldata)

counts_mrna <- round(counts_mrna)

dds_LIHC <- DESeqDataSetFromMatrix(countData = counts_mrna, colData = coldata, design = ~condition

dds <- DESeq(dds_LIHC, parallel = T

resultsNames(dds)

res <- results(dds, name = "condition_tumor_vs_normal") %>%

as.data.frame() %>% #转换成 data.frame

na.omit() %>% #去掉含有 NA 的结果

rownames_to_column("gene_id") %>%

arrange(padj) #再按照 adj p 值并排序

head(res)

write_tsv(res, "2.diff//DEseq2_all_Res.txt")

#hot

library(EnhancedVolcano)

EnhancedVolcano(res,

lab = rownames(res),

x = 'log2FoldChange',

y = 'pvalue')

EnhancedVolcano(res,

lab = rownames(res),

x = 'log2FoldChange',

y = 'pvalue',

title = 'Disease versus Normal',

subtitle = NULL,

caption = NULL)

#GO enrichment analysis

rm(list = ls())

library(tidyverse)

library(clusterProfiler)

library(AnnotationDbi)

library(GOplot)

library(ggplot2)

gsym.fc <- read_tsv("2.diff//DEseq2_diff_Res.xls") %>%

dplyr::select(1,3)

gsym.id <- bitr(gsym.fc$gene_id, fromType = "SYMBOL", toType = "ENTREZID", OrgDb = "org.Hs.eg.db")

gsym.dat <- gsym.fc %>%

dplyr::select(SYMBOL = gene_id,logFC = log2FoldChange) %>%

inner_join(gsym.id,"SYMBOL") %>%

na.omit()

ego <- enrichGO(gene = gsym.dat$ENTREZID, OrgDb = org.Hs.eg.db, ont = "All", pvalueCutoff = 0.05, qvalueCutoff = 0.05)

#KEGG enrichment analysis

kk <- enrichKEGG(gene = gene, organism = "hsa", keyType = "kegg", pvalueCutoff = 0.05, pAdjustMethod = "BH", qvalueCutoff =0.5)

upsetplot(kk) + ggtitle("upsetplot for KEGG")

dotplot(kk)+ scale_color_continuous(type = "viridis") +

ggtitle("dotplot for KEGG")+

aes(shape = Count > 10)

#GSEA enrichment

lapply(c('clusterProfiler','enrichplot','patchwork'),

function(x) {library(x, character.only = T)})

data(geneList, package="DOSE")

#4312 8318 10874 55143 55388 991

#4.572613 4.514594 4.418218 4.144075 3.876258 3.677857

class(geneList)

#[1] "numeric"

kk2 <- gseKEGG(geneList = geneList,

organism = 'hsa',

nPerm = 10000,

minGSSize = 10,

maxGSSize = 200,

pvalueCutoff = 0.05,

pAdjustMethod = "none" )

lapply(c('org.Hs.eg.db','stringr','dplyr'),

function(x) {library(x, character.only = T)})

load(file = 'step2-output.Rdata')

head(deg)

# logFC AveExpr t P.Value adj.P.Val

# COL11A1 7.235897 9.241369 13.66359 1.499441e-14 3.500895e-10

# ZIC2 5.658879 7.917121 13.26803 3.244100e-14 3.787162e-10

# PGM5-AS1 -3.626344 7.758838 -12.66304 1.090802e-13 6.546434e-10

# PGM5 -3.020465 10.639885 -12.59561 1.251776e-13 6.546434e-10

# ANGPTL1 -4.141375 9.283651 -12.53414 1.419781e-13 6.546434e-10

# SHOX2 -5.304161 8.434325 -12.45164 1.682311e-13 6.546434e-10

# B

# COL11A1 22.87130

# ZIC2 22.15323

# PGM5-AS1 21.01867

# PGM5 20.88943

# ANGPTL1 20.77110

# SHOX2 20.61157

logFC_t=1.5

deg$g=ifelse(deg$P.Value>0.05,'stable',

ifelse( deg$logFC > logFC_t,'UP',

ifelse( deg$logFC < -logFC_t,'DOWN','stable') )

)

table(deg$g)

#DOWN stable UP

# 762 21771 815

# 转成ENTREZID

deg$symbol=rownames(deg)

df <- bitr(unique(deg$symbol), fromType = "SYMBOL",

toType = c( "ENTREZID"),

OrgDb = org.Hs.eg.db)

DEG=deg

DEG=merge(DEG,df,by.y='SYMBOL',by.x='symbol')

data_all_sort <- DEG %>%

arrange(desc(logFC))

geneList = data_all_sort$logFC #把foldchange按照从大到小提取出来

names(geneList) <- data_all_sort$ENTREZID #给上面提取的foldchange加上对应上ENTREZID

head(geneList)

#1301 57214 7546 92196 389336 23657

#7.235897 6.273463 5.658879 5.341371 4.958484 4.957802

class(kk2)

#[1] "gseaResult"

#attr(,"package")

#[1] "DOSE"

# 结果保存在kk2@result

colnames(kk2@result)

# [1] "ID" "Description" "setSize"

# [4] "enrichmentScore" "NES" "pvalue"

# [7] "p.adjust" "qvalues" "rank"

# [10] "leading_edge" "core_enrichment"

kegg_result <- as.data.frame(kk2)

rownames(kk2@result)[head(order(kk2@result$enrichmentScore))]

#[1] "hsa00360" "hsa04710" "hsa00350" "hsa00650" "hsa00982" "hsa04974"

gseaplot2(kk2, geneSetID = rownames(kk2@result)[head(order(kk2@result$enrichmentScore))])+

gseaplot2(kk2, geneSetID = rownames(kk2@result)[tail(order(kk2@result$enrichmentScore))])

ridgeplot(kk2, 10)
