# Supplementary material for: USP20 is a predictor of poor prognosis in colorectal cancer and associated with lymph node metastasis, immune infiltration and chemotherapy resistance
Source: Front Oncol. 2023 Feb 16;13:1023292. doi: 10.3389/fonc.2023.1023292 (PMC9978104; doi:10.3389/fonc.2023.1023292)
Supplement: Supplementary Figure 1 — The USP20 expression in CRC from GEO database (GSE32323); [file DataSheet_1.zip › Supplementary Material/Data Sheet 2.DOCX]

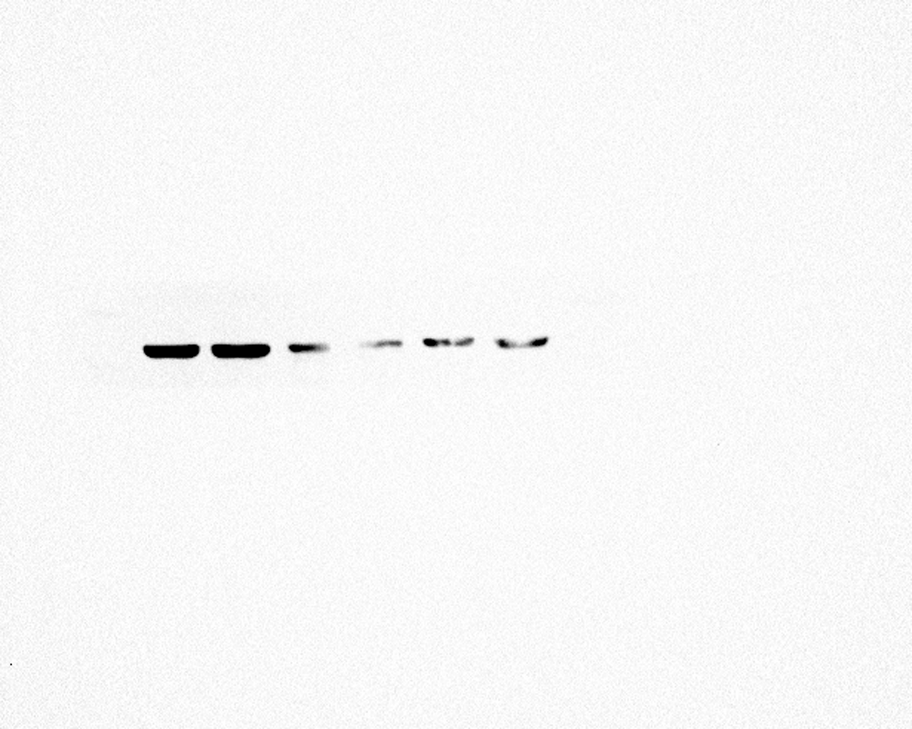


Figure8(A)：bactin.

Note: The six spiked wells are SW480 cells Treatment order is USP20 overexpression plasmid and control plasmid , repeated three times.


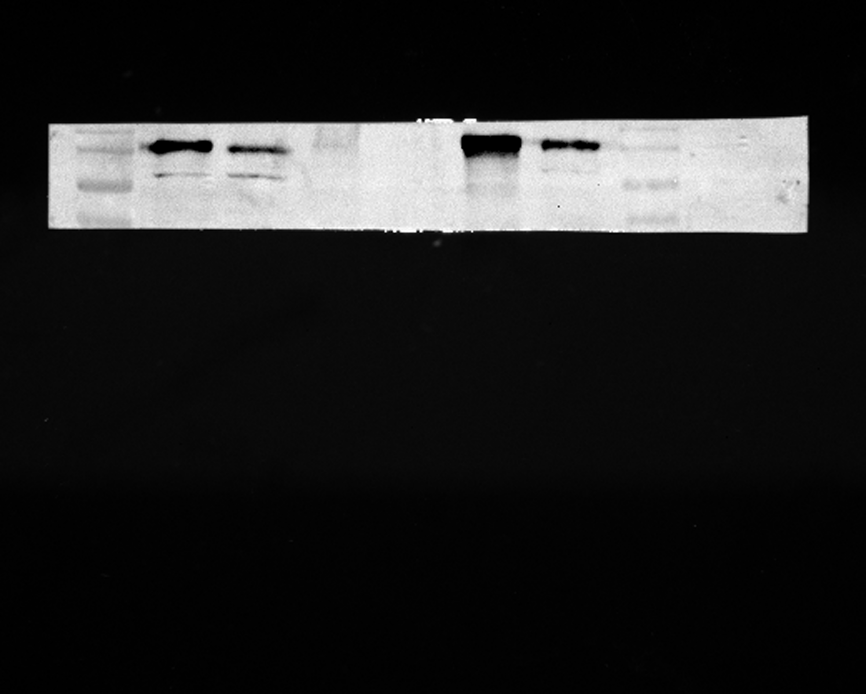


Figure8(A)：USP20.

Note: The six spiked wells are SW480 cells Treatment order is USP20 overexpression plasmid and control plasmid , repeated three times.


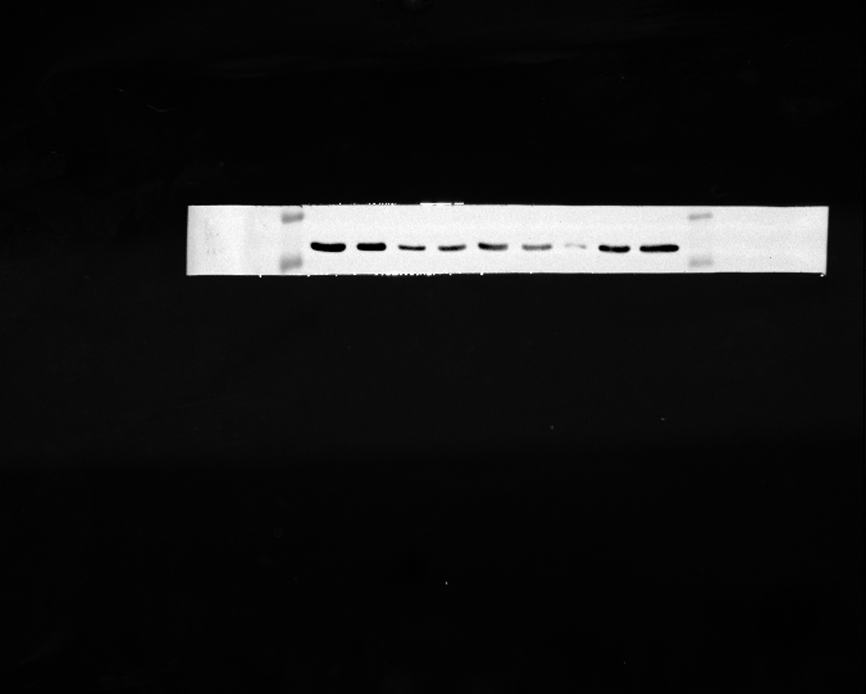


Figure8(A)：bactin.

Note: The six spiked wells are SW480 cells Treatment order is USP20 overexpression plasmid and control plasmid , repeated three times. This is a repeated experiment.


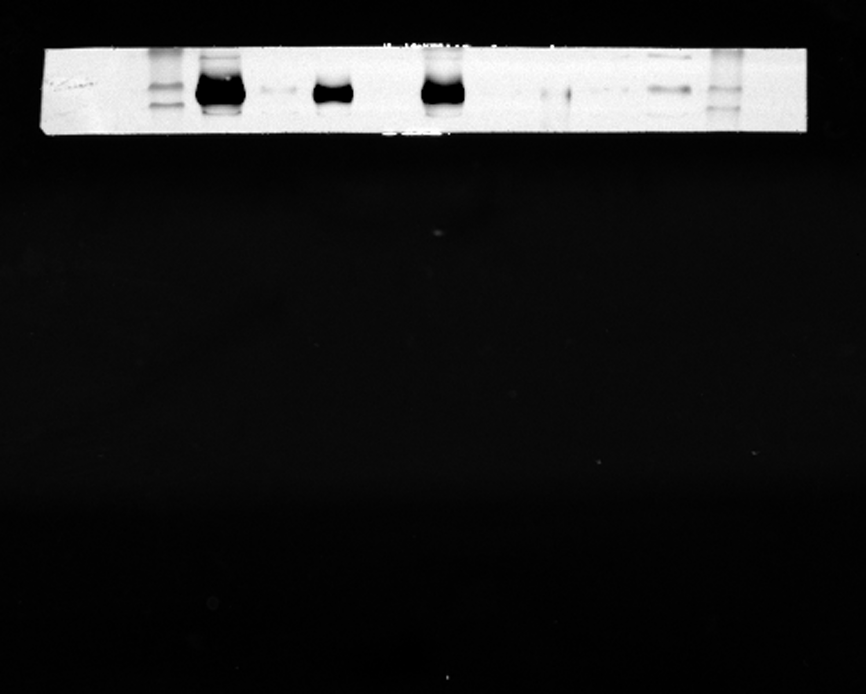


Figure8(A)：USP20

Note: The six spiked wells are SW480 cells Treatment order is USP20 overexpression plasmid and control plasmid , repeated three times. This is a repeated experiment.


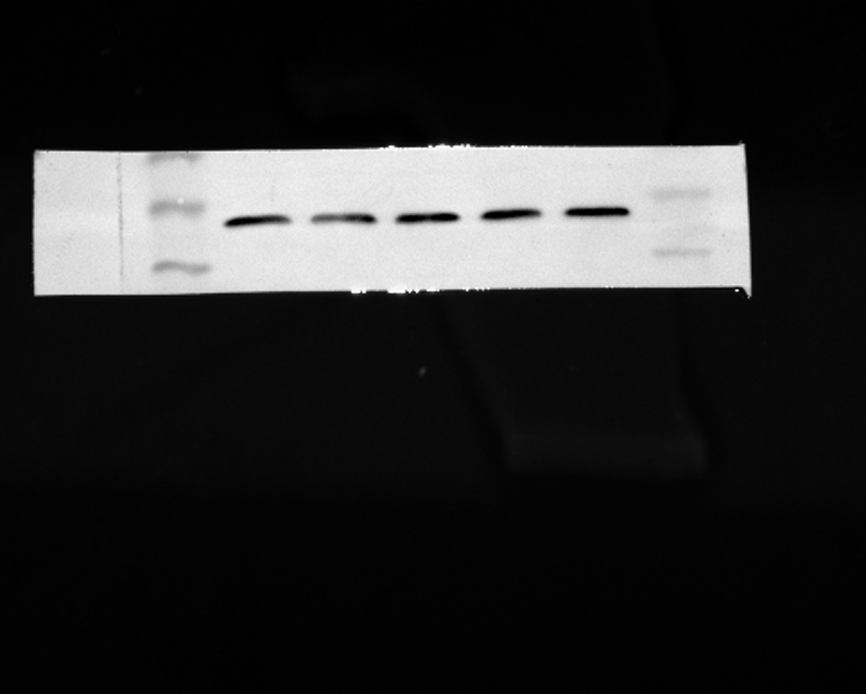


Figure8(H)：GAPDH

Note: The first six spiked wells are SW480 cells Treatment order is NC-siRNA，siRNA-3-1816，siRNA-2-1310，siRNA-1-208，NC-siRNA.


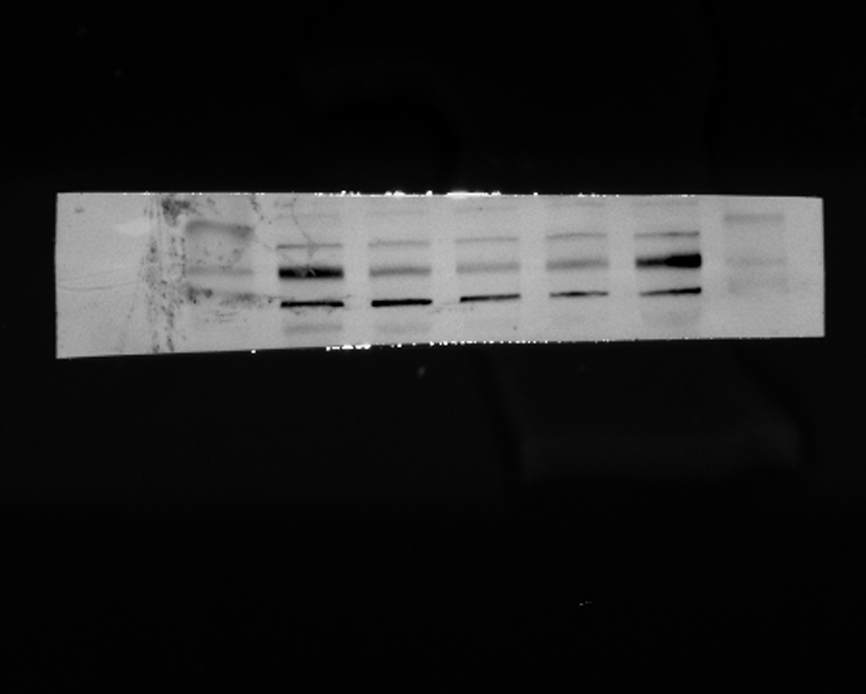


Figure8(H)：USP20

The first six spiked wells are SW480 cells Treatment order is NC-siRNA，siRNA-3-1816，siRNA-2-1310，siRNA-1-208，NC-siRNA.
