# Supplementary figures and images for: USP20 is a predictor of poor prognosis in colorectal cancer and associated with lymph node metastasis, immune infiltration and chemotherapy resistance
Source: Front Oncol. 2023 Feb 16;13:1023292. doi: 10.3389/fonc.2023.1023292 (PMC9978104; doi:10.3389/fonc.2023.1023292)

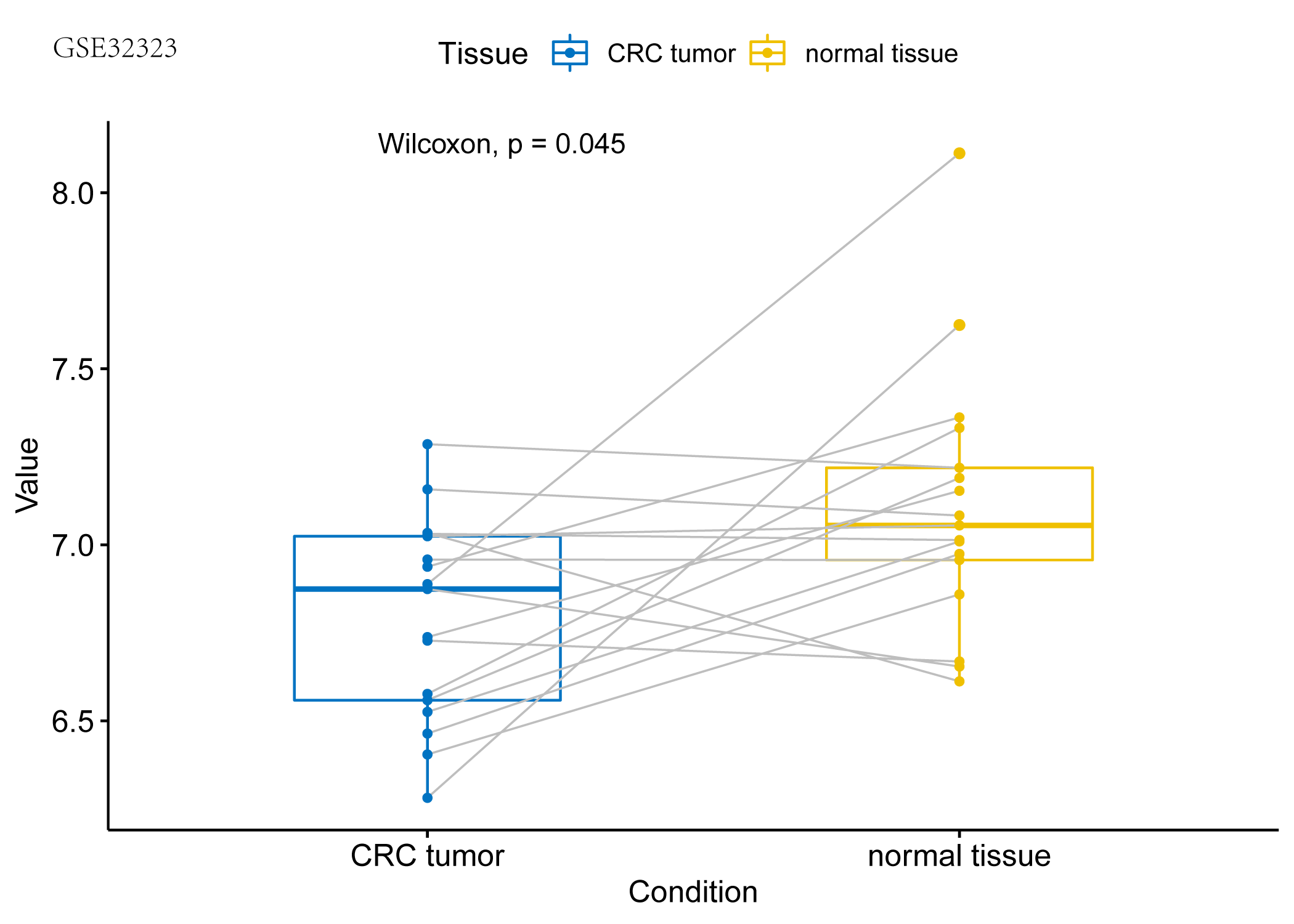

Supplement: Supplementary Figure 1 — The USP20 expression in CRC from GEO database (GSE32323); [file DataSheet_1.zip › Supplementary Material/Supplement Figure 1.TIF]

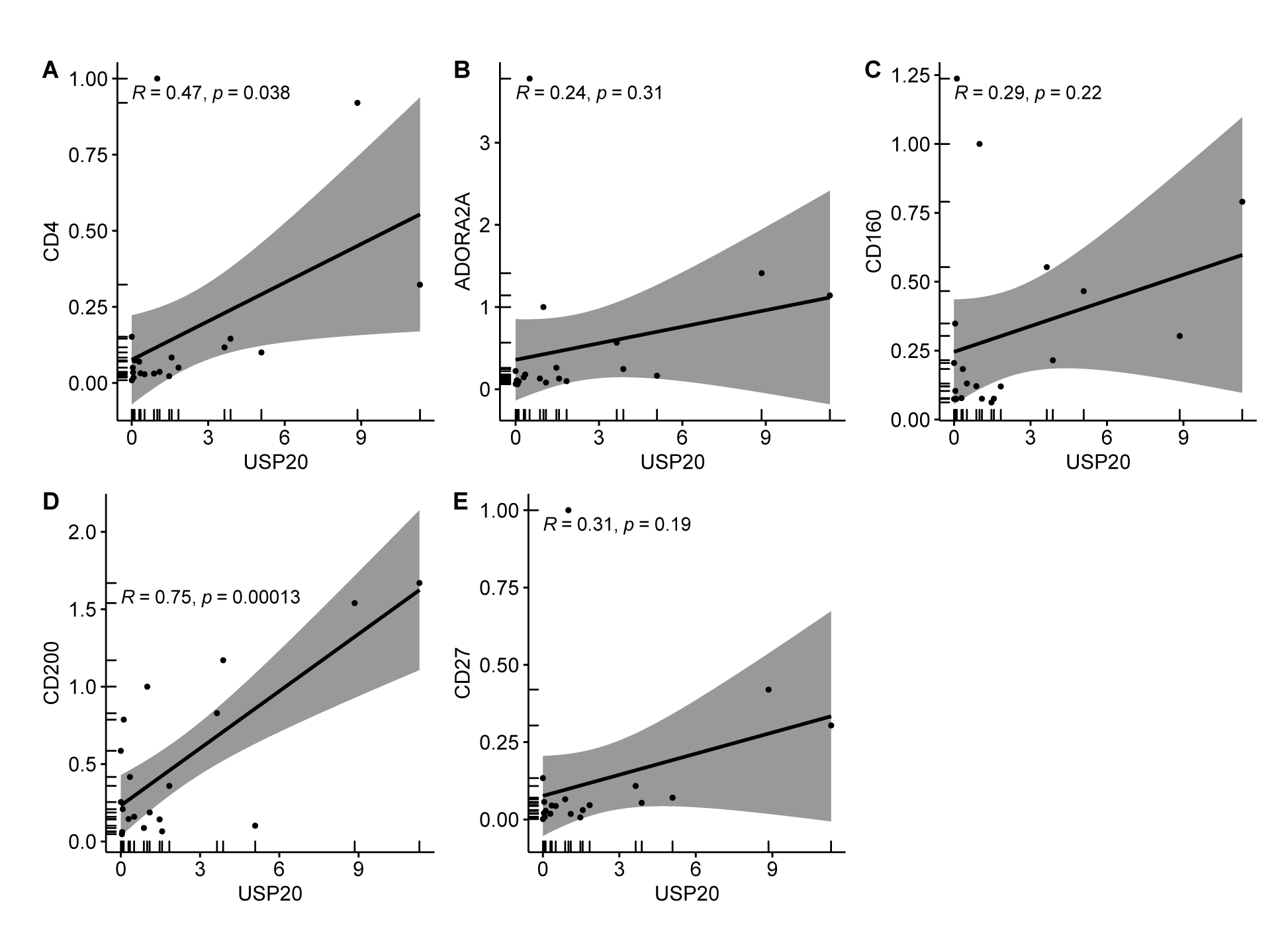

Supplement: Supplementary Figure 1 — The USP20 expression in CRC from GEO database (GSE32323); [file DataSheet_1.zip › Supplementary Material/Supplement Figure 2.TIF]
